# Controlling the Order–Disorder Transition Temperature through Anion Substitution in CuCr*X*_2_ (*X* = S, Se, Te)

**DOI:** 10.1021/acs.chemmater.5c01384

**Published:** 2025-08-21

**Authors:** Md Towhidur Rahman, Noah P. Holzapfel, Kamil Ciesielski, Weeam Guetari, Eric Toberer, Veronica Augustyn, Alexandra Zevalkink

**Affiliations:** 1 Department of Mechanical Engineering, 3078 Michigan State University, East Lansing, Michigan 48824, United States; 2 Department of Chemical Engineering and Materials Science, 3078 Michigan State University, East Lansing, Michigan 48824, United States; 3 Department of Materials Science and Engineering, 6798 North Carolina State University, Raleigh, North Carolina 27695, United States; 4 Department of Physics, 3557 Colorado School of Mines, Golden, Colorado 80401, United States

## Abstract

In solid-state ion conductors, order–disorder transitions often govern the onset of superionic behavior, making them a key target for tuning ionic mobility. Layered *A*Cr*X*_2_ (*A* = Ag, Cu; *X* = Se, S) chalcogenides have high ionic conductivity enabled by cation site disorder associated with a high-temperature phase. In this work, we investigated alloying with S or Te at the anion site in CuCrSe_2_ and the impact that alloying has on the degree of cation disorder and the temperature of the order–disorder transition. We prepared a series of polycrystalline CuCrSe_2‑*x*_Te_*x*_ (*x* = 0, 0.1, 0.15, 0.175) and CuCrSe_2‑*y*_S_*y*_ (*y* = 0, 0.1, 0.25, 0.5, 0.75, 1.0, 2.0) compounds by solid-state synthesis. X-ray diffraction analysis confirmed that the S–Se system exhibits complete solubility, whereas Te substitution at the anion site in CuCrSe_2_ is limited to *x* = 0.15. Variable temperature X-ray diffraction and thermal diffusivity measurements were conducted to track the order–disorder and superionic transition temperature (*T*_c_) of the compounds. The transition temperature was found to be highly composition-dependent, exhibiting a decreasing trend with the incorporation of larger anions; CuCrSe_1.85_Te_0.15_ had the lowest *T*_c_ at 282 K, which is the lowest reported *T*_c_ to date for bulk samples in this crystal structure type. We also investigated the elastic properties and speed of sound in the CuCrSe_2‑*x*_Te_*x*_ series as functions of composition and temperature. We show that the samples soften sharply as the anion size increased. As a function of temperature, we see only a small inflection of the temperature coefficient of elasticity, d*C*_ij_/d*T*, at the order–disorder phase transition, confirming prior findings that long-wavelength acoustic phonons are largely unaffected by the phase transition. Thermoelectric (TE) characterizations were also performed, revealing that the TE figure of merit of the compounds remains nearly unchanged at high temperatures (493 K). These findings demonstrate that tuning interatomic distances and bond stiffness through the anion site alloying can effectively tailor the behavior of solid-state ionic conductors.

## Graphical abstract

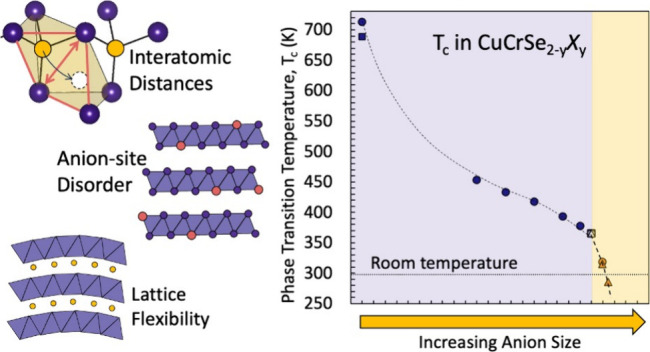

## Introduction

In the pursuit of sustainable energy storage, the development of high-performance solid-state electrolytes is crucial, particularly those capable of fast ion conduction under ambient conditions. Superionic conductors (SIC), with their exceptionally high ionic mobility, promise significant advancements for enhancing battery safety and longevity.^1–3^ Layered *A*Cr*X*_2_ compounds (*A* = Cu, Ag and *X* = S, Se) with the trigonal crystal structure shown in Figure 1a have gained much interest recently both as superionic conductors and as potential thermoelectric materials, as they exhibit fast ion transport and low lattice thermal conductivity. Both properties stem from the same structural characteristics including soft bonding, high anharmonicity, and cation site disorder.^4,5^ The structure of CuCrSe_2_ is defined by layers of edge-sharing CrSe_6_ octahedra, alternating with layers of Cu in the tetrahedral sites.^6–8^ In the room-temperature-ordered phase, Cu atoms occupy every other tetrahedral sites. As the temperature approaches the order–disorder phase transition, however, Cu atoms move freely into adjacent empty tetrahedral spaces, eventually resulting in 50% occupancy of all available tetrahedral sites. Note that the disorder increases gradually with increasing temperature, becoming fully disordered only at the phase transition temperature (*T*_c_). At this point, the symmetry increases from *R*3*m* to *R*3̅*m*. In the *A*Cr*X*_2_ compounds, the order–disorder transition, which is marked by a significant increase in the ion mobility, occurs at elevated temperatures (350–700 K). Among unalloyed *A*Cr*X*_2_ compounds, CuCrSe_2_ has the lowest *T*_c_ at 365 K,^9–11^ while CuCrS_2_ has the highest *T*_c_ at 688 K.^10^ It should be noted that few studies on 2D AgCrS_2_ compounds^12,13^ recently reported room temperature superionic behavior; however, in the bulk, this is only observed above 673 K.

**Figure 1:**
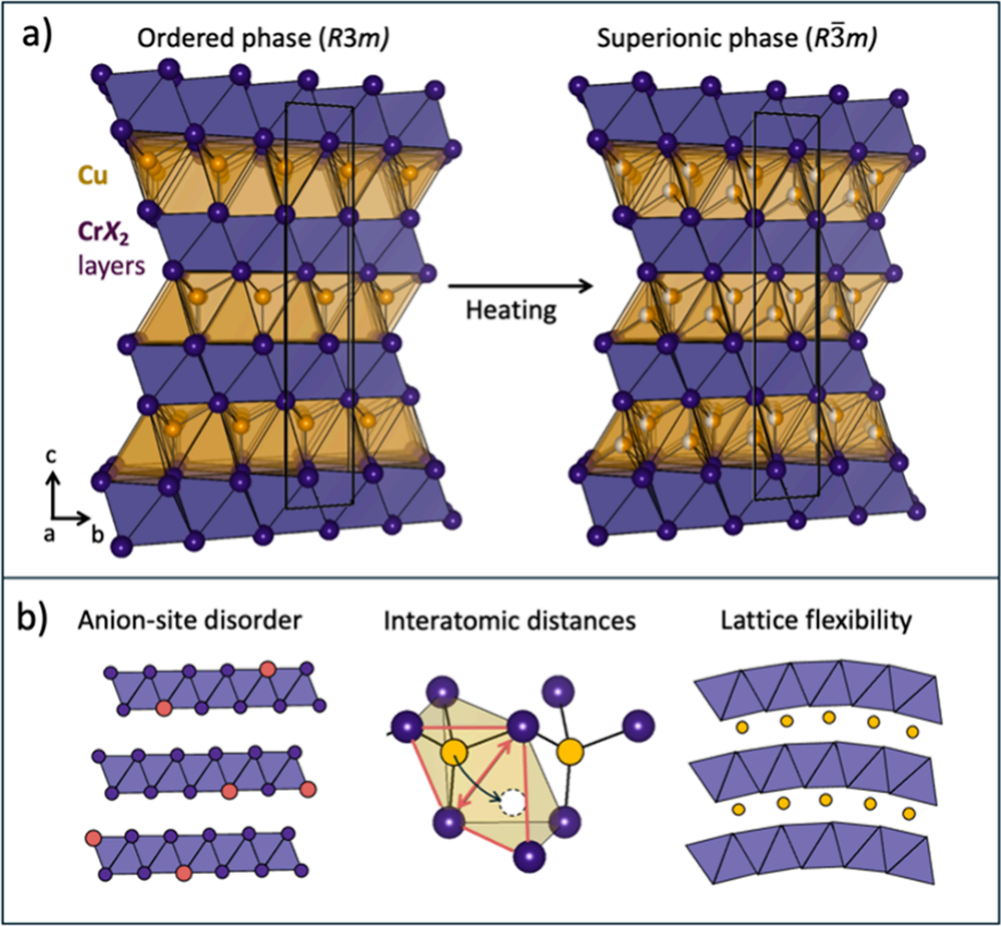
(a) Layered crystal structure of CuCrSe_2_. As temperature increases, the Cu ions move into the empty tetrahedral site, increasing symmetry from *R*3*m* → R3̅m. (b) Alloying on the anion site impacts the transition temperature by increasing disorder, changing the bottleneck size for Cu jumps, and controlling the overall lattice flexibility.

Having the ability to systematically control the order–disorder temperature, *T*_c_, of superionic conductors–including *T*_c_ of *A*Cr*X*_2_ compounds–is a central goal for advancing both the fundamental science and practical applications of superionic conductors. In general, the order–disorder phase transition temperature in superionic conductors is influenced by a combination of structural, chemical, and dynamic factors, as shown schematically in Figure 1b. The crystal structure and lattice geometry play a critical role,^14,15^ as open frameworks or larger interstitial voids reduce the energy barriers for ion migration, thus lowering *T*_c_.^16,17^ Likewise, the size and polarizability of the ions play a role in determining the electrostatic potential landscape. Larger, more polarizable ions and their associated soft, anharmonic vibrational modes typically lead to lower transition temperatures.^4,18^ Site disorder on the anionic framework is an additional important factor, as increased configurational entropy (Δ*S*) has been shown to lead to a decrease in phase transition temperature.^19–23^ External factors can also modulate the lattice environment; for instance, applied high pressure initially increases the energy barrier and, therefore, *T*_c_.^24,25^

Alloying serves as a crucial strategy for tuning various factors that govern the order–disorder/superionic transition temperature (*T*_c_).^26,27^ In the *A*Cr*X*_2_ system, alloying has been explored to a limited extent on both the cation or anion sites.^28,29^ Cation site alloying is often challenging due to limited miscibility. The AgCrSe_2_–CuCrSe_2_ system shows near-zero miscibility at room temperature.^28^ More recently, Izumi et al.^30^ demonstrated minimal Cu and Au solubility (<3%) in AgCrSe_2_ on the basis of synchrotron diffraction. They did find, however, that both larger and smaller cation substitutions led to a decrease in the *T*_c_ for AgCrS_2_. In contrast, alloying on the anion site may offer greater flexibility; at least in the case of the S–Se system, complete solubility has been reported.^5^ However, the influence of the anion site alloying on *T*_c_ remains completely unexplored. Moreover, Te substitution in CuCrSe_2_ has not been attempted.

Given these knowledge gaps, there is a significant scope for a systematic investigation into anion substitution in *A*Cr*X*_2_ compounds and its effect on *T*_c_. In this study, we examine the solid solubility of polycrystalline CuCrSe_2‑*x*_Te_*x*_ (*x* = 0, 0.1, 0.15, 0.175) and CuCrSe_2‑*y*_S_*y*_ (*y* = 0, 0.1, 0.25, 0.5, 0.75, 1.0, 2.0) compounds. The CuCr*X*_2_ system was selected due to its lower transition temperatures compared with other compounds in the series, making it an ideal candidate for a detailed study. As highlighted in Figure 1b, we explored these compounds with a focus on anion site disorder, anion size, and lattice flexibility and the impact of these factors on the order–disorder phase transition temperature.

## Materials and Methods

### Synthesis

Polycrystalline CuCrSe_2‑*x*_Te_*x*_ (*x* = 0.1, 0.15, 0.175) and CuCrSe_2‑*y*_S_*y*_ (*y* = 0.1, 0.25, 0.5, 0.75, 1.0, 2.0) samples were synthesized by a solid-state reaction followed by spark plasma sintering (SPS). The synthesis of the end member, CuCrSe_2_, was described in ref 11, and the same sample was used in the current study. For alloy synthesis, stoichiometric amounts of Cu (powder, Alfa Aesar, 99.9% purity), Cr (chips, Sigma-Aldrich, 99.995% purity), Se (shot, Alfa Aesar, 99.999% purity), S (shot, Alfa Aesar, 99.999% purity), or Te (shot, Alfa Aesar, 99.999% purity) were weighed inside an argon-filled glovebox. Then, the elements (5 g/batch) were filled into quartz ampules (16 mm outer diameter) and taken outside the glovebox for sealing. The ampules were sealed under static vacuum pressure less than 10^–4^ Torr and inserted into the furnace. The samples were heated to 773 K at a 0.5 K/min heating rate, held at 773 K for 12 h, then heated to 1273 K at the same heating rate, held for 24 h, and finally, cooled to room temperature over 12 h. Afterward, the obtained ingots were taken inside the glovebox and sealed in stainless steel SPEX vials with three 7/16” stainless steel ball bearings. The samples were then ball-milled for 6 min using a SPEX Sampleprep 8000D. The SPEX vials were then brought back into the glovebox, opened, and the powders were scraped out to recover >90% of the initial load. The obtained fine powders were then loaded into graphite dies (≈1.5 g/batch) and sintered in an SPS press at 1023 K for 30 min under 40 MPa pressure. The resulting consolidated cylindrical pellets were polished to obtain flat and parallel surfaces for characterization. All samples were found to be >95% of their theoretical density.

### X-ray Diffraction

The phase purity of the solid cylindrical polycrystalline samples was checked with X-ray diffraction (XRD) using a Rigaku SMARTLAB diffractometer operated at 40 kV and 44 mA with a Cu Kα radiation source. The obtained XRD patterns were analyzed to determine secondary phases, and lattice parameters were obtained with Rietveld refinement using Rigaku PDXL-2 software.

### Variable Temperature X-ray Diffraction

Variable temperature X-ray diffraction (VT-XRD) data were collected in Bragg–Brentano geometry on a PANalytical Empyrean diffractometer (45 kV, 40 mA, sealed Cu X-ray tube, Kα_1_/Kα_2_ λ = 1.5406 Å/1.5444 Å) equipped with a TTK-450 (Anton Paar) nonambient sample stage and a PIXcel1D position sensitive detector. The solid cylindrical polycrystalline sample was adhered to the sample stage with a heat conducting paste to mitigate thermal gradients between the stage and the sample. Once placed on the stage, the instrument was calibrated with Z- and Ω-alignment scans to account for the height differences of the pellets. All measurements were performed under a flowing nitrogen gas. In order to cool below room temperature, a continuous flow of liquid nitrogen was cycled through the sample stage from an Oxford Cryosystems dewar with an Anton Paar LNC Nitrogen suction pump unit. Samples were cooled and heated at rates of 1–2 °C/min. Continuous XRD patterns were collected over a narrow angular range (10–40 °2θ) with a low integration time leading to approximately one scan per minute. Upon reaching each temperature set point, a single 15 min scan was collected over the same angular range. All diffraction measurements had step sizes of 0.02 °2θ.

### Microstructural Analysis

Secondary electron and backscattered electron images of the flat parallel surface of the samples were collected using a JEOL 6610LV scanning electron microscope with a tungsten emitter at the Michigan State University Center for Advanced Microscopy. EDS (energy dispersive X-ray spectroscopy) mapping and line scanning were conducted using Oxford EDS systems.

### Transport Property Measurement

Thermal diffusivity (*D*) of the samples was measured using a NETZSCH Light Flash Apparatus (LFA) 467 HyperFlash. The cylindrical samples with flat parallel surfaces were first coated with graphite. Argon was used as a purge and protective gas. The system was vacuumed and purged three times before the experiment. Consecutive heating and cooling cycles were run within the temperature range from 300 to 723 K with five degree steps. Thermal conductivity (κ_total_) was calculated using the equation κ_total_ = ϱ × *D* × *C*_p_, where *D* is thermal diffusivity, ϱ is the geometric density, and *C*_p_ is the constant pressure heat capacity obtained with the Dulong-Petit approximation. The resistivity and Hall effect were collected on a custom-built apparatus described in ref 31 using Van der Pauw technique. The current supplied was 150 mA, while the magnetic field amounted to 1 T. The Seebeck coefficient was also studied on a home-built apparatus described in ref 32. For both electronic measurements, heating and cooling curves were collected to ensure a lack of thermal hysteresis.

### Resonant Ultrasound Spectroscopy

Resonant ultrasound spectra at room temperature were collected using a RUS008 system from Alamo Creek Engineering and RUSpy, an open-sourced software. Temperature-dependent data from 260 to 345 K were collected at 5 K increments with an RTC004 system from Alamo Creek Engineering, which employs thermoelectric heater/cooler. The instrumental setup and work procedure are discussed by Migliori et al.^33,34^ The samples were mounted between two piezoelectric transducers with the diagonally opposite corners making contact to approximate the free boundary conditions. Then, the samples were excited at frequencies ranging from 50 to 450 kHz to determine the resonance peaks. RUScal software^35^ was used to determine the elastic moduli for each sample by inverse numerical analysis using resonance peaks from the spectrum. As the samples were polycrystalline, they are considered isotropic, and the elastic tensor can be fully described by just two independent terms: C_11_ and C_44_. Young’s modulus (*Y*), shear modulus (*G*), bulk modulus (*B*), Poisson’s ratio (μ), longitudinal velocity (*v*_L_), and shear velocity (*v*_S_) were then obtained from C_11_ and C_44_.^36^

## Results and Discussions

### Phase Purity and Lattice Parameters

Figure 2a shows the room temperature X-ray diffraction patterns for CuCrSe_2‑*x*_Te_*x*_ (*x* = 0, 0.1, 0.15, 0.175) and CuCrSe_2‑*y*_S_*y*_ (*y* = 0, 0.1, 0.25, 0.5, 0.75, 1.0, 2.0) samples densified using spark plasma sintering. The XRD patterns show that all samples crystallize in either the ordered *R*3*m* or disordered *R*3̅*m* structure type. The two variants can be most readily distinguished by the intensity of the (015) reflection. Ding et al.^10^ measured the temperature dependence of the intensity of selected Bragg peaks for *A*Cr*X*_2_ samples with *A* = Cu, Ag and *X* = S, Se. They reported that the (015) peak is gradually suppressed with increasing temperature and disappears in the superionic phase. Here, we use the (015) peak intensity as an effective order parameter. In Figure 2b, we see that the peaks corresponding to the (015) reflection both decrease in intensity and shift to the left with increasing anion size. Figure 3c shows the normalized intensity of the (015) reflection plotted as a function of composition for the CuCrSe_2‑*y*_S_*y*_ and CuCrSe_2‑*x*_Te_*x*_ series, where rapid suppression of intensity can be observed for the Te-alloyed samples. Partial suppression of the (015) peak is indicative of partial disorder of the Cu ions. The (015) peak is almost completely suppressed in CuCrSe_1.85_Te_0.15_, which is indicative of the sample being in the fully disordered phase at room temperature and suggesting that it has *T*_c_ below room temperature.

**Figure 2:**
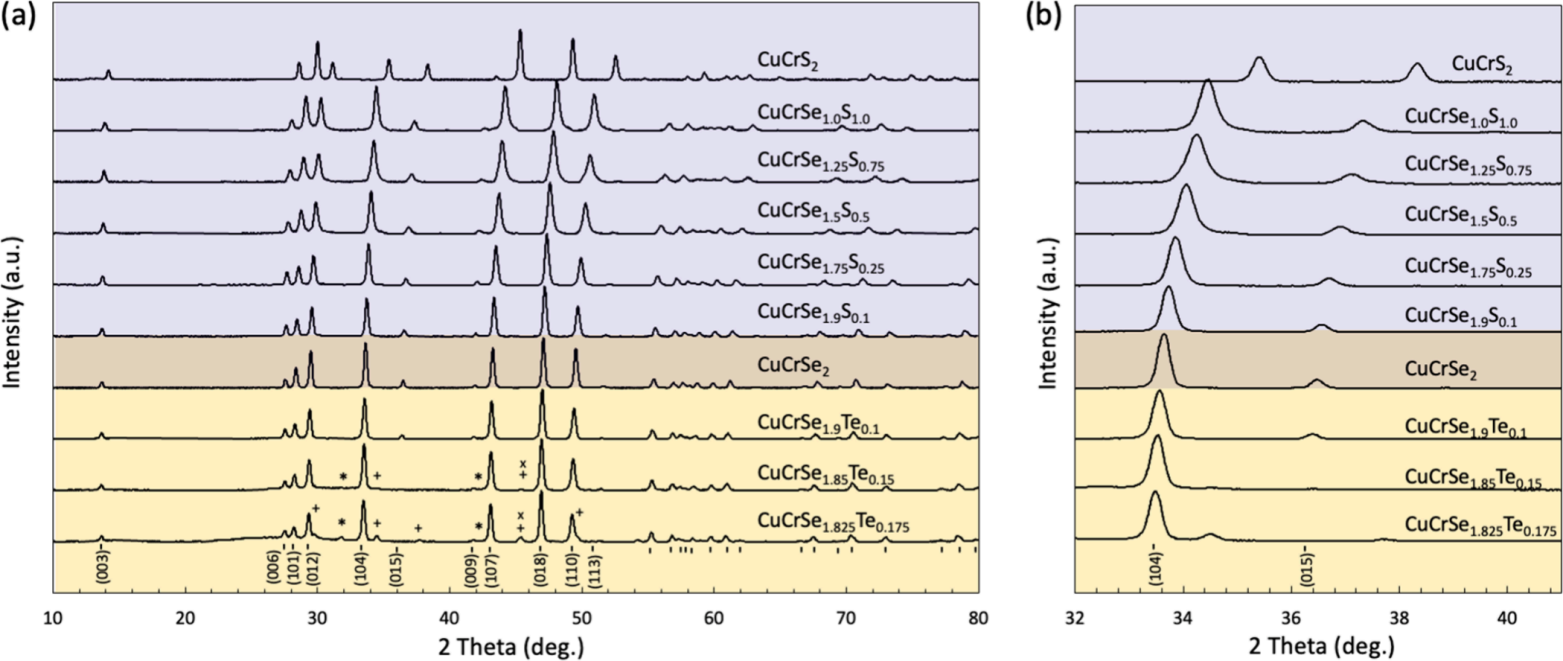
(a) X-ray diffraction pattern at room temperature for CuCrSe_2‑*x*_Te_*x*_ (*x* = 0, 0.1, 0.15, 0.175) and CuCrSe_2‑*y*_S_*y*_ (*y* = 0, 0.1, 0.25, 0.5, 0.75, 1.0, 2.0) compounds. The blue and yellow regions separate the S-alloyed and Te-alloyed samples. Impurity phases found only in the Te-rich samples are Cr_3_Se_4_ (*), Cu_2_Te (x), and CuCr_2_Se_4_ (+). (b) (104) and (015) peaks, showing shift toward larger 2-theta values as larger anion fraction decreases (from Te-rich toward S-rich compositions) in the samples. The (015) reflection intensity decreases as we move from S-rich composition to Se-rich ones. With an increasing Te content, the (015) peak intensity further goes down and is observed to be almost suppressed in the *x* = 0.15 sample.

**Figure 3:**
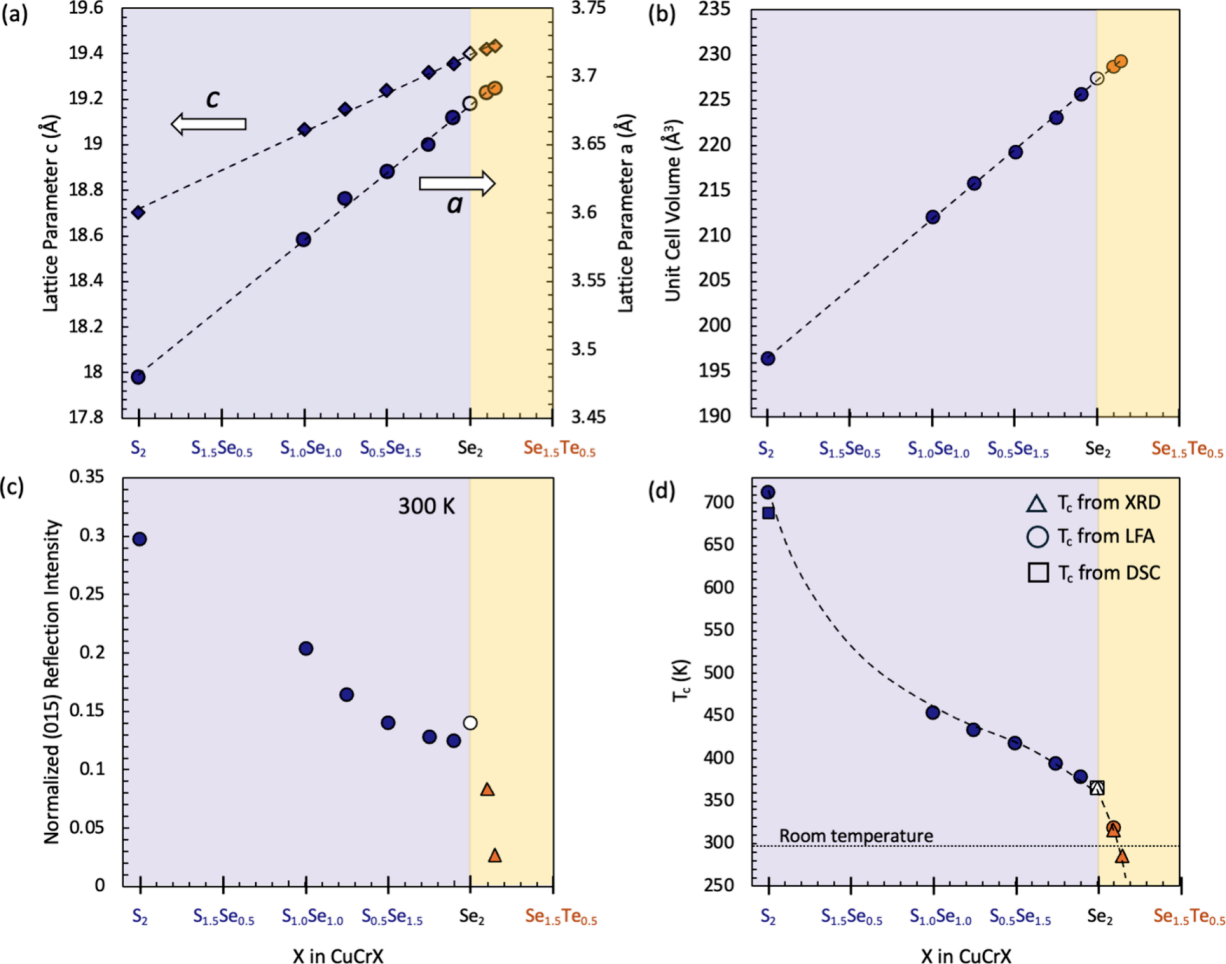
(a) Lattice parameters *a* and *c* and (b) unit cell volumes are plotted as a function of composition in the CuCrSe_2‑*x*_Te_*x*_ and CuCrSe_2‑*y*_S_*y*_ series, showing a linear trend. (c) Normalized intensity of (015) reflection at room temperature (300 K) is plotted as a function of composition, where the intensity decreases gradually as larger anions are substituted. Smaller intensity signifies a more disordered system, and with Te substitution at the anion site, the normalized intensity is significantly suppressed, almost to zero. The highly suppressed (015) reflection intensity of the CuCrSe_1.85_Te_0.15_ sample suggests a fully disordered system. (d) Superionic transition temperature (*T*_c_) as a function of composition, obtained from DSC,^10^ VT-XRD, and thermal diffusivity measurements. A nonlinear decrease in *T*_c_ with increasing anion size can be observed.

With increasing anion radius (S → Se → Te), the XRD peaks of the primary phase gradually shift to lower angles, indicating increased lattice parameters. An increase in lattice parameter is expected as the ionic radius of Te^2–^ (2.21 Å) is larger than that of Se^2–^ (1.98 Å) and S^2–^ (1.84 Å).^37–39^ Figure 3a,b shows the linear increase in lattice parameters *a* and *c* and the unit cell volume, *V*, obtained from Rietveld refinement. Rietveld refinements and different profiles are shown for all samples in Figure S1, and Table S1 lists the so-obtained lattice parameters. This linear trend points to the formation of a solid solution in the system for the complete CuCrSe_2‑*y*_S_*y*_ series and up to at least *x* = 0.15 for the CuCrSe_2‑*x*_Te_*x*_ series. There is a very small amount (<3%) of secondary phases of Cr_3_Se_4_, Cu_2_Te, and CuCr_2_Se_4_ observed in the *x* = 0.15 sample, while for *x* = 0.175, a more significant amount of the secondary phases (Cr_3_Se_4_, Cu_2_Te, and CuCr_2_Se_4_) starts to emerge. Microstructural analysis using EDS (energy dispersive X-ray spectroscopy) mapping (Figures S2–S4) and line scanning (Figure S5) on BSE (backscattered electron) images of the flat parallel surface of the CuCrSe_2‑*x*_Te_*x*_ (*x* = 0, 0.1, 0.15) samples also shows traces of Cr_3_Se_4_ and Cu_2_Te in CuCrSe_1.85_Te_0.15_ (Figure S4). The larger impurity concentration in *x* = 0.175 suggests that the solubility of Te has been exceeded and that excess Te is precipitated primarily as Cu_2_Te. Note that CuCrTe_2_ is not isostructural with CuCrSe_2_, and therefore, it was not expected to form a complete solid solution. In the following discussion, we only include data on samples with the Te content up to *x* = 0.15.

### Impact of Alloying on Phase Transition Temperature, *T*_c_

Figure 3d shows an overview of the *T*_c_ as a function of composition in the CuCrSe_2‑*x*_Te_*x*_ and CuCrSe_2‑*y*_S_*y*_ series, obtained from either variable temperature X-ray diffraction (VT-XRD), light flash analysis (LFA) thermal diffusivity, or both. We also compared previously reported *T*_c_ obtained by DSC measurements for CuCrSe_2_ and CuCrS_2_.^10^ As we introduce larger atoms at the anion sites in the CuCr*X*_2_ system, the transition temperature decreases, dipping below room temperature only for the most Te-rich sample, CuCrSe_1.85_Te_0.15_.

We collected continuous and stepwise VT-XRD patterns for samples in the CuCrSe_2‑*x*_Te_*x*_ (*x* = 0, 0.1, 0.15) series. Figure 4 shows the results for *x* = 0.15, and Figure S6 shows the results for samples with *x* = 0.10 and *x* = 0. For the *x* = 0.15 sample, data were collected continuously from 300 to 243 K (1st cooling cycle), then from 243 K up to 373 K (1st heating cycle), and finally from 373 to 243 K (second cooling cycle). As expected, the most significant change in the diffraction pattern upon heating/cooling is the intensity of the (015) reflection at around 36.4 °2θ. This is highlighted in the right panel of Figure 4. The (015) reflection is visible only at temperatures below 282 K, and the change in intensity is reversible. This confirms that the order–disorder phase transition temperature was below room temperature for CuCrSe_1.85_Te_0.15_ (*T*_c_ = 282 K). For the CuCrSe_2_ and CuCrSe_1.85_Te_0.1_ samples, based on the change in the (015) reflection intensity, the phase transition temperatures were 365 and 315 K, respectively. Figure S7 shows the longer, 15 min scans collected at predetermined temperature intervals below and above *T*_c_ for the three CuCrSe_2‑*x*_Te_*x*_ (*x* = 0, 0.1, 0.15) samples.

**Figure 4:**
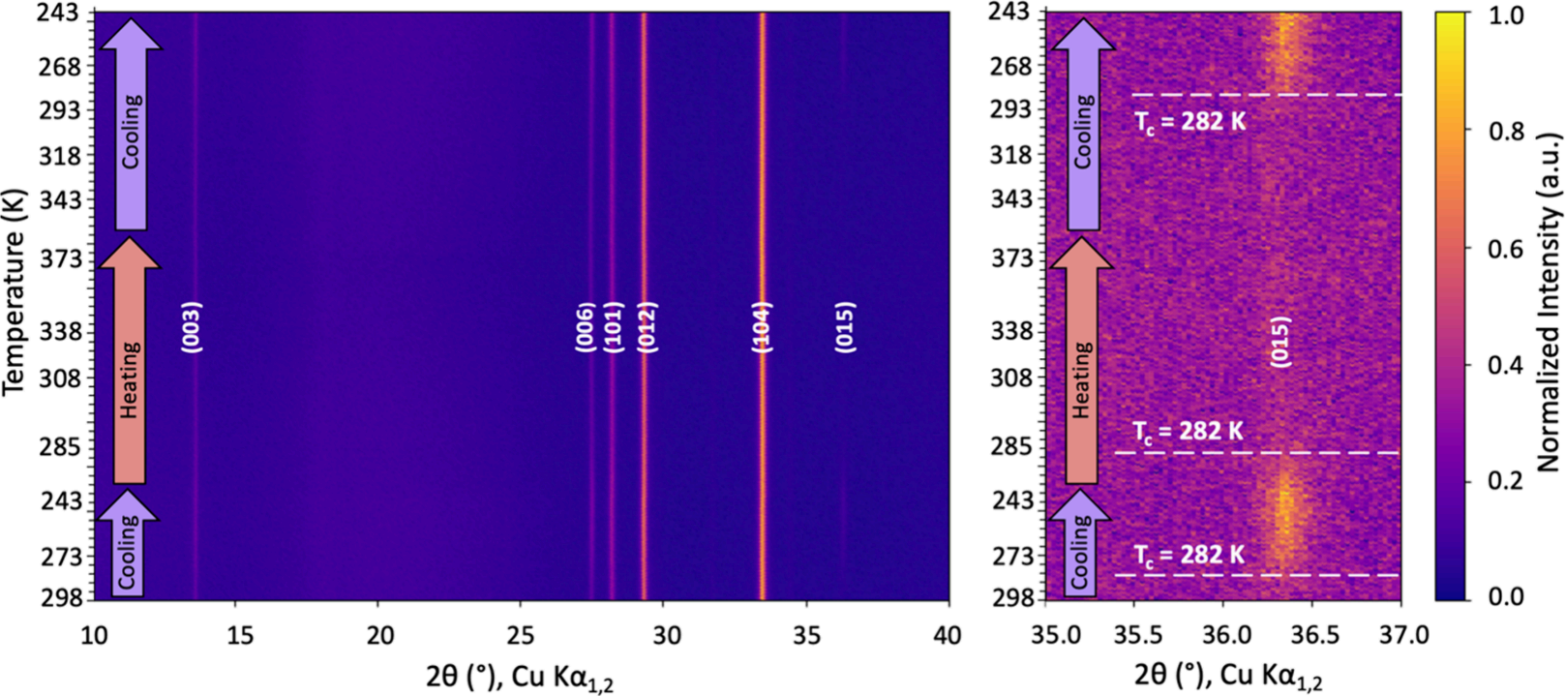
Contour plot of the variable temperature continuous X-ray diffraction for CuCrSe_1.85_Te_0.15_ (left panel). The contrast in normalized intensity shows the appearance and disappearance of the (015) peak at 282 K (right panel), indicating a superionic phase transition at that temperature. The blue and red arrows indicate the cooling and heating cycles, respectively. XRD data was collected continuously at an approximately 1 scan/min rate.

Figure 5 shows thermal diffusivity (*D*) collected at 5 K intervals for CuCrSe_2‑*y*_S_*y*_ (*y* = 0, 0.1, 0.25, 0.5, 0.75, 1.0, 2.0) and CuCrSe_2‑*x*_Te_*x*_ (*x* = 0, 0.1, 0.15, 0.175) compounds. An inverted peak, corresponding to the latent heat absorbed during the phase transition, can be observed for each composition except for CuCrSe_1.85_Te_0.15_. The total thermal conductivity of the Te samples, estimated using κ_total_ = ϱ × *D* × *C*_p_, is shown in Figure S8a, and its electronic (κ_e_ = *LT*/ρ) and lattice (κ_l_ = κ_total_ – κ_e_) components are presented in Figure S11, where *L* is the Lorenz number determined from Seebeck coefficient, *L* = 1.5 + exp(*−*|*S*|/116).^40^ Here, however, we decided to focus on raw diffusivity data since the temperature at which this peak is observed can be used as a relatively accurate (±5 K) measurement of *T*_c_. As we move from S-rich composition to Se-rich, the inverted peak shifts toward lower temperature, which is shown in Figure 5a. A similar shift occurs as we move from CuCrSe_2_ to Te-containing compositions as shown in Figure 5b. All of these indicate that *T*_c_ decreases with increasing anion size. For CuCrSe_1.85_Te_0.15_, no peak was observed, indicating that the sample is already in a fully disordered phase, in agreement with the VT-XRD data. The insets show thermal diffusivity as a function of composition at room temperature. With increased disorder in the system, thermal diffusivity, and thus thermal conductivity, is expectedly observed to decrease, reaching the lowest point for the most disordered system. In the CuCrSe_2‑*y*_S_*y*_ series, the lowest thermal diffusivity was found in CuCrSe_1.0_S_1.0_ at room temperature.

**Figure 5:**
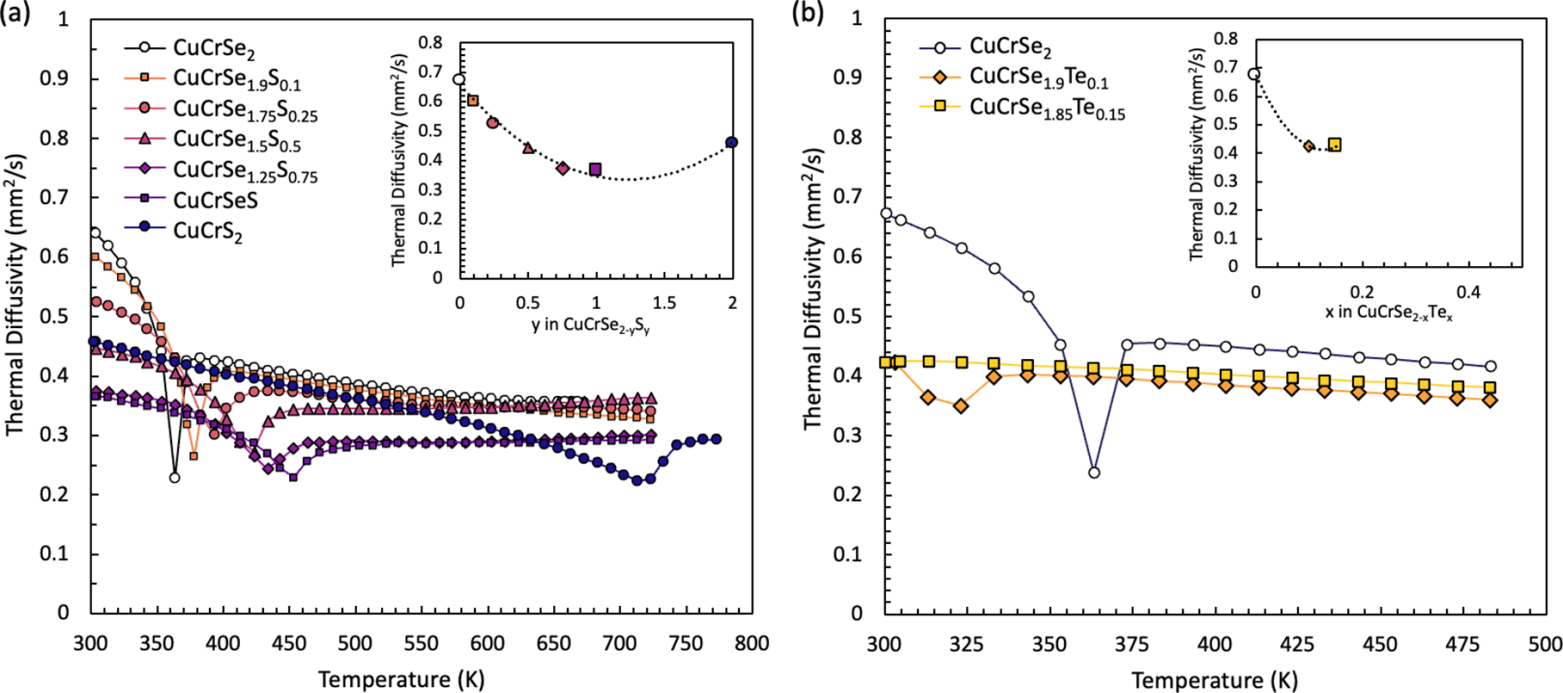
Thermal diffusivities (*D*) for CuCrSe_2‑*y*_S_*y*_ (*y* = 0, 0.1, 0.25, 0.5, 0.75, 1.0, 2.0) and CuCrSe_2‑*x*_Te_*x*_ (*x* = 0, 0.1, 0.15, 0.175) compounds. With a decreasing S content (a) and increasing Te content (b), the inverted peaks, which correspond to latent heat absorption, shift to lower temperature. This indicates that superionic transition temperature (*T*_c_) decreases as larger atoms increase at the anion sites. The insets show room temperature thermal diffusivities as a function of composition. For CuCrSe_2‑*y*_S_*y*,_ the lowest thermal conductivity can be observed at the highest disorder sample (CuCrSe_1.0_S_1.0_).

To test the stability of *T*_c_ in the Te-alloyed samples and, thus, to ensure that the Te substitution on the Se site is not metastable, we measured thermal diffusivity multiple times on the same sample and also on multiple samples (as shown in Figure S14). The *T*_c_ obtained from the measurements was consistently below room temperature (i.e., outside the measurement range) in the *x* = 0.15 sample, and the *T*_c_ remained consistent in the *x* = 0.1 sample, which suggests that the Te substitution at the Se site is stable.

### Thermoelectric Transport Properties in the CuCrSe_2‑*x*_Te_*x*_ Series

The thermoelectric transport properties of the end-members CuCrS_2_ and CuCrSe_2_ have been previously characterized by us and by others.^5,6,8,41–44^ While the sulfide was found to be electrically insulating due to an extremely low carrier concentration, CuCrSe_2_ was previously shown to be a promising thermoelectric material (if one disregards potential issues associated with the ionic transport). In the current study, we measured the electronic transport properties of the CuCrSe_2‑*x*_Te_*x*_ series (*x* = 0, 0.1, 0.15) and found minimal changes in properties, with no overall improvement in the thermoelectric figure of merit, *zT = S* ^*2*^ *T*/ρκ.^45,46^ These measurements are reported in the Supporting Information Section. Figure S8 shows the thermal and electronic properties of CuCrSe_2‑*x*_Te_*x*_ compounds. All samples show degenerate p-type semiconducting behavior. The carrier concentration, shown in Figure S9a, varies between 1 × 10^20^ and 3 × 10^20^ cm^–3^, but it does not exhibit a clear trend with respect to the Te content in the system. In contrast, in Figure S9b, the electronic mobility shows a strong decrease with increased Te concentration in the CuCrSe_2‑*x*_Te_*x*_ series, which can likely be attributed to both increasing point defects and impurity phase concentration. Figure S10 shows the Seebeck coefficient, mobility, and *zT* as a function of carrier concentration (*n*_h_), compared to a single parabolic band model assuming acoustic deformation scattering^47^ and an effective mass of m* = 1.23 m_o_. Figure S12 presents transport data during heating and cooling cycles for the CuCrSe_2‑*x*_Te_*x*_ series, which exhibits a slight hysteresis. To evaluate the reproducibility of the electronic transport measurements, successive measurements were conducted on the Te-alloyed samples. As shown in Figure S13, the Seebeck coefficient values are highly consistent across measurements, whereas the resistivity data exhibit a variation of approximately 20%.

### Elastic Properties and Speed of Sound

Figure 6 shows the room temperature Young’s modulus (*Y*) and shear modulus (*G*) for the CuCrSe_2‑*y*_S_*y*_ and CuCrSe_2‑*x*_Te_*x*_ series of samples. As the anion size increases (S → Se → Te), both the Y and G can be seen to decrease. The rate of softening with respect to composition appears to be somewhat higher in the Se–Te series when compared to that of the S–Se series. This is likely due to the larger size delta between Te and Se than between Se and S. Overall, we see an ∼30% reduction in stiffness across the series of samples, with increasing anion size. Room temperature values of the elastic moduli, as well as Poisson’s ratio (μ), longitudinal velocity (*v*_L_), and shear velocity (*v*_S_) are presented in Table S2 for all compositions.

**Figure 6:**
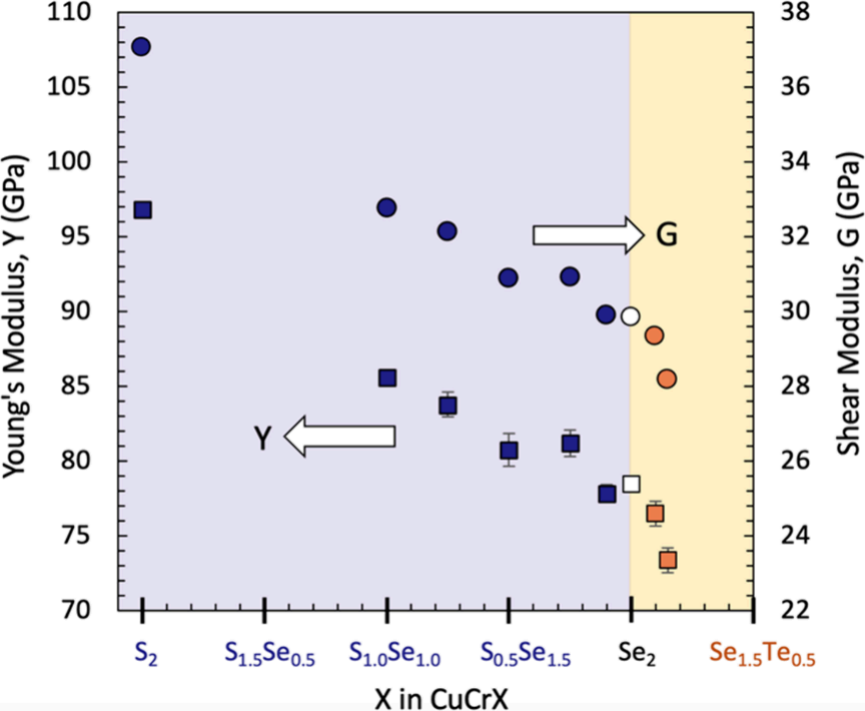
Room temperature Young’s modulus (squares) and shear modulus (circles) as a function of composition. The elastic moduli decrease as the anion sites are filled with larger atoms, indicating the softening of bonds. The rate of softening, however, varies between the S–Se and Se–Te series due to the larger size delta between Se and Te. Error bars based on goodness-of-fit are shown for all of the data points. For G, the error bars are smaller than the symbols.

It is well-known that elastic stiffness is an important factor controlling the energy barrier for ionic diffusion.^18^ In contrast, the role that lattice stiffness plays in determining the transition temperature for order–disorder transitions is rarely discussed. As in any thermodynamically governed phase transition, the temperature of the order–disorder transition in CuCr*X*_2_ is determined by the competition of the enthalpy change, Δ*H*, and the entropy change, Δ*S*, caused by the phase transition. Δ*H* is the term favoring the ordered phase, while Δ*S* favors the disordered phase. We postulate that an increased anion size in the *A*Cr*X*_2_ system has two important effects: first, reducing the lattice stiffness increases the vibrational entropy by decreasing the average phonon energy. While this should affect both the ordered and disordered phases,^48^ it is reasonable to expect that the vibrational entropy increase in the disordered phase is larger,^49^ which may be a key reason that a softer lattice leads to a reduction in the order–disorder phase transition temperature. Second, larger anions promote the disordered phase by expanding the in-plane lattice parameters, thus decreasing the electrostatic repulsion between Cu cations occupying neighboring sites (e.g., in the disordered phase). Indeed, this seems to be a general trend in Ag or Cu conducting compounds with cation order–disorder transitions; lower *T*_c_ can generally be correlated with larger anions.^50,51^ Finally, although the increasing lattice parameters and decreasing elastic constants explain the overall decrease in *T*_c_ with increasing anion size, they do not explain the nonmonotonic trend in *T*_c_. For samples in the S–Se series, we see that alloyed samples have *T*_c_ suppressed below a simple rule of mixtures. This disorder-induced reduction of *T*_c_ has been seen in other studies as well^21^ and may stem from local strain and randomized local bonding environments, which lower the enthalpy to be gained from cation ordering.

The elastic moduli of CuCrSe_2‑*x*_Te_*x*_ samples with *x* = 0–0.15 were also measured as a function of temperature. For these measurements, we were limited to a temperature range of 260–345 K, and we, therefore, focused our efforts on samples with *T*_c_ in that range. The temperature-dependent evolution of Y and G throughout the experimental temperature range for one heating cycle is presented in Figure 7. For the Te-substituted compositions, we measured multiple samples to check the consistency of our data. Additional data are shown in Figures S15 and S16, including heating and cooling cycles for all samples and measurements of duplicate samples, to confirm reproducibility and reversibility.

**Figure 7:**
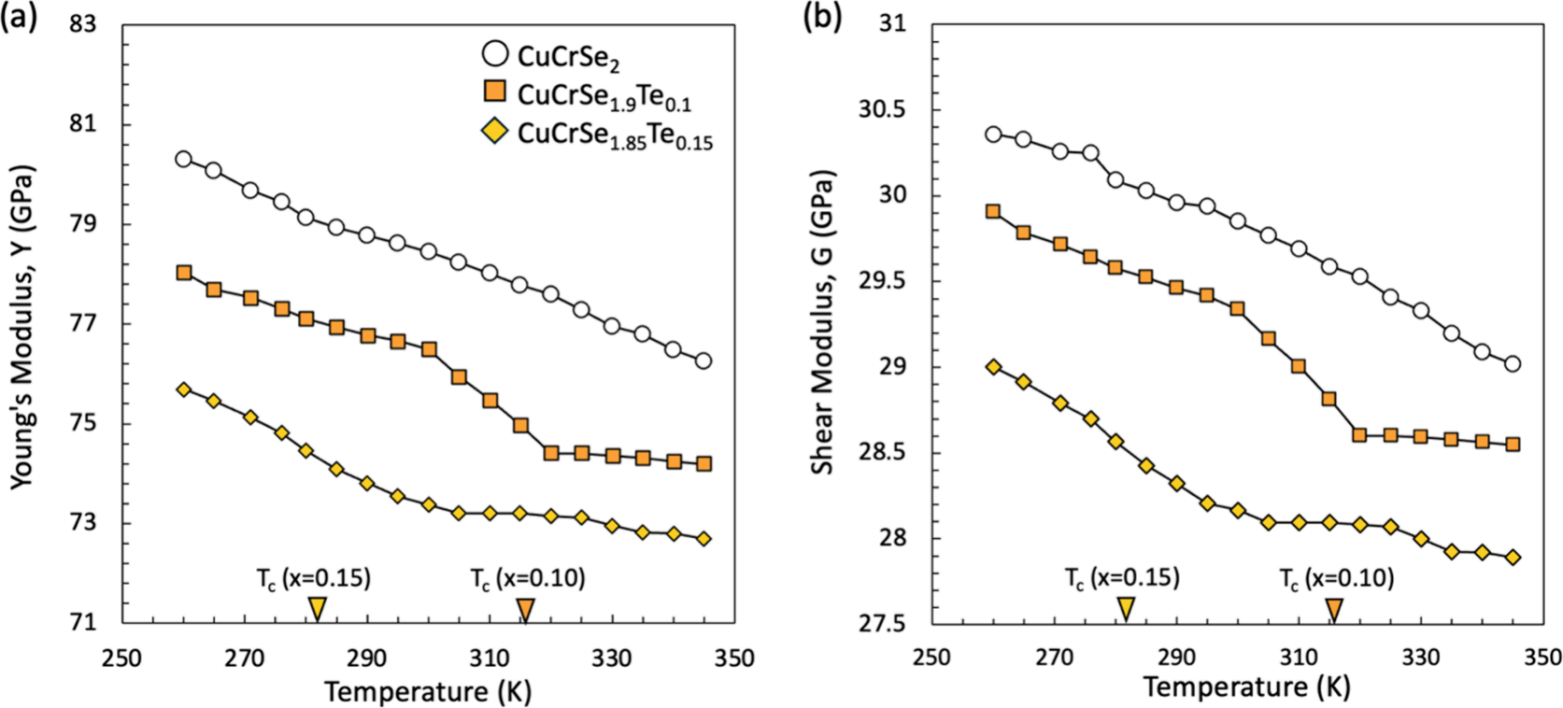
Temperature-dependent (a) Young’s modulus and (b) shear modulus for CuCrSe_2‑*x*_Te_*x*_ (*x* = 0, 0.1, 0.15) compounds. The orange and yellow arrows indicate the superionic transition temperatures obtained from VT-XRD for the CuCrSe_1.9_Te_0.1_ and CuCrSe_1.85_Te_0.15_ samples. Temperature coefficient of elastic constants (d*C*/d*T*) changes for CuCrSe_1.9_Te_0.1_ (≈320 K) and CuCrSe_1.85_Te_0.15_ (≈285 K) but remained almost constant for CuCrSe_2_.

For CuCrSe_2_, which has *T*_c_ (365 K) outside of the measurement range, the elastic constants exhibit a smooth decrease with an increasing temperature, i.e., the temperature coefficients of elasticity, d*Y*/d*T* and d*G*/d*T*, remain constant with temperature. This is a typical behavior, as bonds soften upon heating due to thermal expansion. In contrast, a sudden decrease in the temperature coefficient of elasticity was observed for both CuCrSe_1.9_Te_0.1_ (≈320 K) and CuCrSe_1.85_Te_0.15_ (≈285 K) at temperatures that are in reasonably good agreement with the order–disorder phase transition temperatures obtained from VT-XRD and LFA. To the best of our knowledge, AgI, SrCl_2_, and PbF_2_ are the only few examples of superionic conductors for which the elastic moduli have been measured across cation-sublattice order–disorder phase transition.^52–54^ These compounds also exhibit a discontinuity in the temperature derivative of the elasticity. But notably, consistent with our results for CuCr*X*_2_, the impact is not drastic, and there is no sudden change in stiffness as would be expected for a first order phase transition. It is, therefore, likely that long-wavelength acoustic phonons, which are controlled by elastic properties, would not be dramatically affected by the superionic transition. This is in contrast to the strong impact that the phase transition seems to have on zone-edge acoustic phonons and on optical phonons.^55^

## Conclusions

In this study, we demonstrated complete solubility at the anion site in the CuCrSe_2‑*y*_S_*y*_ series and partial solubility in the CuCrSe_2‑*x*_Te_*x*_ series, as indicated by the precipitation of Te-rich secondary phases above the solubility limit of *x* = 0.15. We showed that the order–disorder transition temperature (*T*_c_) can be tuned via composition: with substitution of larger anions, *T*_c_ decreases and is eventually suppressed to below room temperature with *T*_c_ ≈ 282 K for CuCrSe_1.85_Te_0.15_. This was confirmed through VT-XRD and thermal diffusivity measurements. This effect of anion substitution on *T*_c_ can be explained with alteration of Coulombic interactions, where increasing anion size leads to softer bonds and increased cross sectional area for ion hopping, lowering the activation energy for ion migration. This means that energy required for order–disorder transition can be achieved at relatively lower temperature for compounds with a similar structure but larger atoms at anion sites. Finally, temperature-dependent elasticity measurements demonstrate that the order–disorder transition leads to a change in slope (d*C*_ij_/d*T*), without any discontinuity in stiffness. This suggests that although transverse acoustic phonons may be strongly scattered by cation site disorder, their velocities are not strongly impacted by the phase transition. This work can be extended for further investigations on alloying at both cation and anion sites and the corresponding impact on order–disorder transitions, ionic mobility, as well as thermal transport mechanisms.

## Supplementary Material



## Notes

Rietveld refinement on X-ray diffraction data, SEM images (BSE) including EDS mapping of CuCrSe_2‑*x*_Te_*x*_, variable temperature X-ray diffraction contour plot for CuCrSe_2_ and CuCrSe_1.9_Te_0.1_, thermal and electronic transport properties, corresponding reproducibility and stability test, electronic and lattice thermal conductivity, Pisarenko plots, measurement of elastic properties during heating and cooling cycles, reproducibility of elastic data, and equations for elastic modulus and speed of sound calculation (PDF)
