# Supplementary material for: Controlling the Order–Disorder Transition Temperature through Anion Substitution in CuCrX 2 (X = S, Se, Te)
Source: Chem Mater. 2025 Aug 21;37(17):6718–26. doi: 10.1021/acs.chemmater.5c01384 (PMC12424124; doi:10.1021/acs.chemmater.5c01384)
Supplement: Supplementary file 1 [file cm5c01384_si_001.pdf]

Controlling the Order-Disorder Transition Temperature through Anion Substitution in $CuCrX_2$ (X = S, Se, Te)

Md Towhidur Rahman[1], Noah Holzapfel[3], Kamil Ciesielski[4], Weeam Guetari[2], Eric Toberer[4], Veronica Augustyn[3], Alexandra Zevalkink[2]*

[1] Department of Mechanical Engineering, Michigan State University, East Lansing, MI, USA

[2] Department of Chemical Engineering and Materials Science, Michigan State University, East Lansing, MI, USA

[3] Department of Materials Science and Engineering, North Carolina State University, Raleigh, NC, USA

[4]Department of Physics, Colorado School of Mines, Golden, CO, United States

* Corresponding author: alexzev@msu.edu

Supplementary Information

Lattice Parameters

	a (Å)	b (Å)	c (Å)	Majority Phase	Rwp (%)
$CuCrS_2$	3.4828(5)	3.4828(5)	18.706(2)	R3m	2.49
$CuCrSe_{1.0}S_{1.0}$	3.5836 (3)	3.5836 (3)	19.0667 (10)	R3m	1.83
$CuCrSe_{1.25}S_{0.75}$	3.6060 (2)	3.6060 (2)	19.1640 (7)	R3m	2.5
$CuCrSe_{1.5}S_{0.5}$	3.6275 (10)	3.6275 (10)	19.2419 (2)	R3m	2.21
$CuCrSe_{1.75}S_{0.25}$	3.6519 (5)	3.6519 (5)	19.315(2)	R3m	2.86
$CuCrSe_{1.9}S_{0.1}$	3.6688 (3)	3.6688 (3)	19.3645 (9)	R3m	3.77
$CuCrSe_2$	3.6779 (7)	3.6779 (7)	19.3919 (7)	R3m	3.33
$CuCrSe_{1.9}$ $Te_{0.1}$	3.6875 (8)	3.6875 (8)	19.4155(6)	R3m	3.53
$CuCrSe_{1.85}$ $Te_{0.15}$	3.6912 (6)	3.6912 (6)	19.4292(9)	$R\bar{3}m$	3.87

Table S1: Lattice parameters for $CuCrSe_{2-y}S_y$ and $CuCrSe_{2-x}Te_x$ samples obtained by Rietveld refinement of room temperature powder X-ray diffraction data on polycrystalline pucks.

Rietveld Refinement of X-ray Diffraction Pattern

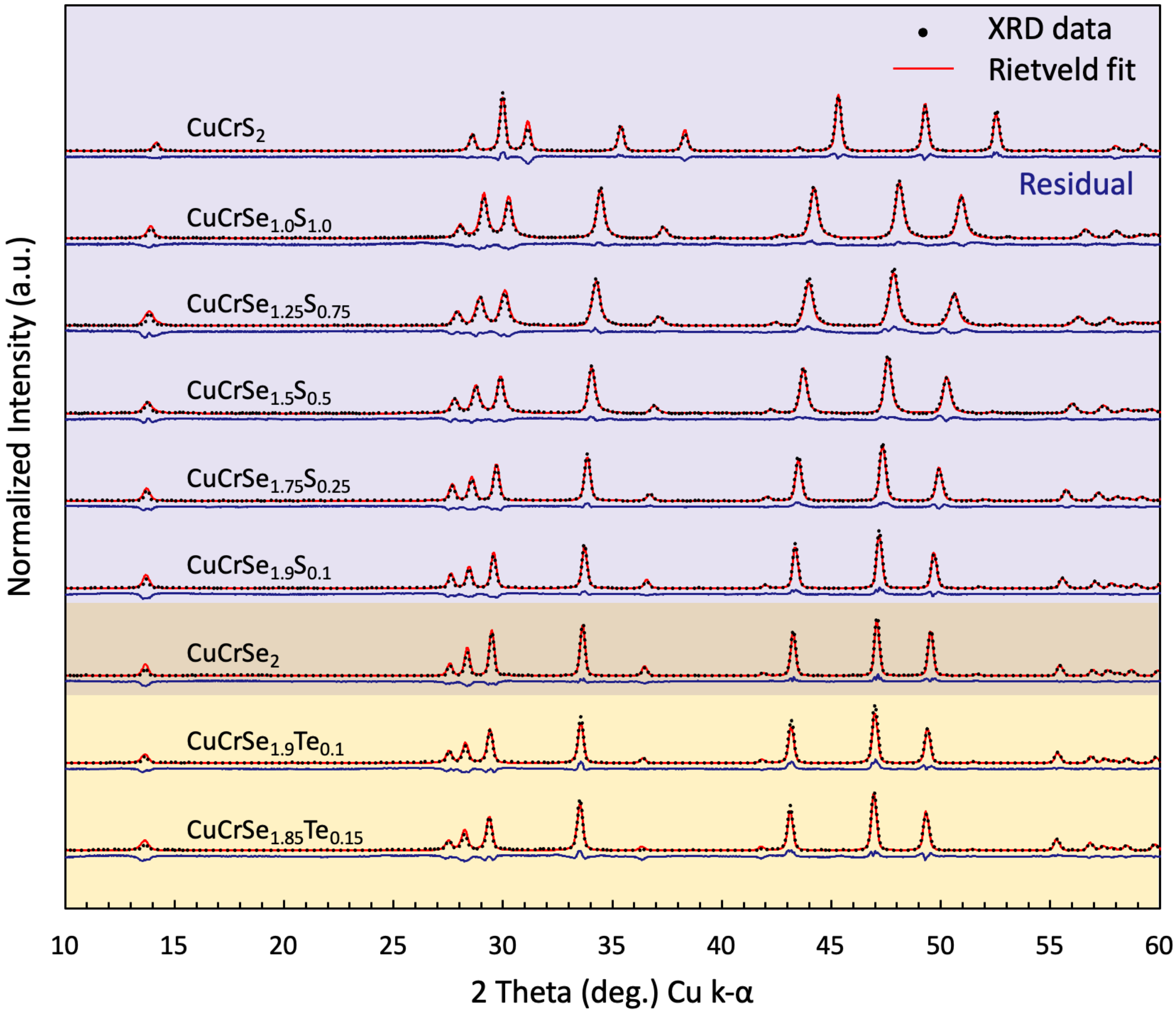

Figure S1: Rietveld fit on X-ray diffraction data on polycrystalline pucks with nominal composition $CuCrSe_{2-x}Te_x$ (x = 0, 0.1, 0.15) and $CuCrSe_{2-y}S_y$ (y = 0, 0.1, 0.25, 0.5, 0.75, 1.0, 2.0). Rietveld refinement was done using PDXL-2 software. Peak shape parameters, lattice parameters, and preferred orientation (001) were refined.

Microstructural Analysis: SEM (BSE) Images of $CuCrSe_{2-x}Te_x$ samples

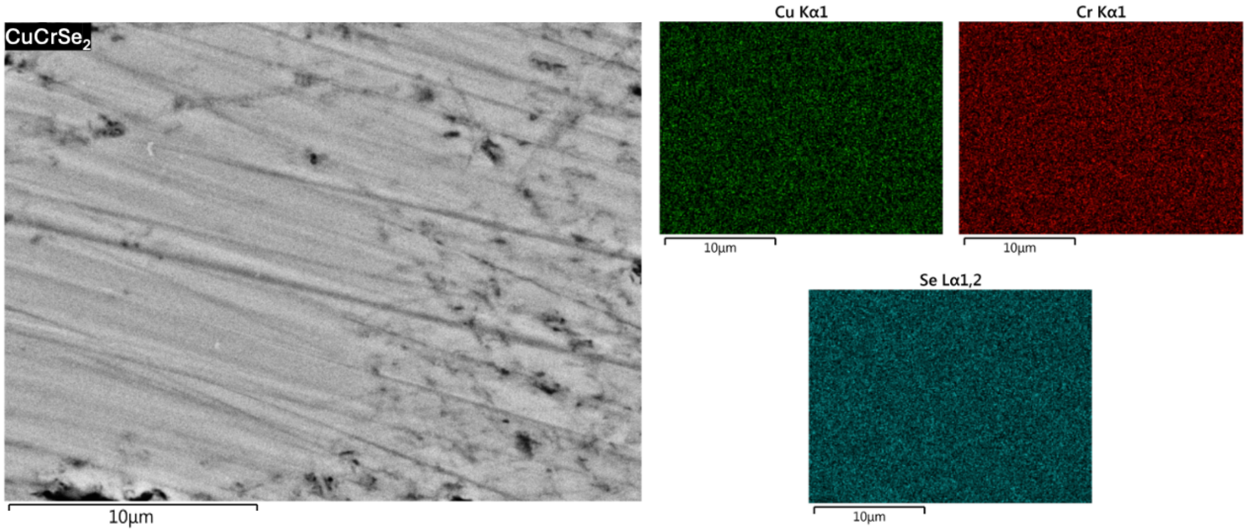

Figure S2: Backscattered electron (BSE) imaging of $CuCrSe_2$ sample, taken on flat parallel surface, polished up to P4000 grit size. The visibly contrasted parallel lines are scratch marks from polishing (left panel). EDS (Energy dispersive X-ray spectroscopy) mapping shows uniform distribution of Cu, Cr and Se across the sample (right panel).

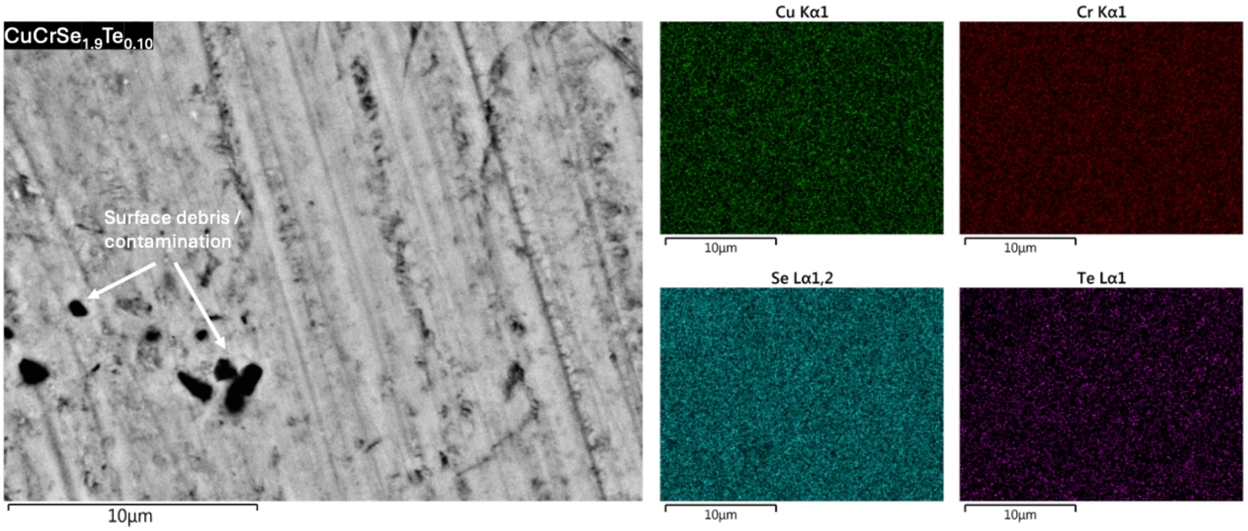

Figure S3: Backscattered electron (BSE) imaging of $CuCrSe_{1.9}Te_{0.1}$ sample, taken on flat parallel surface, polished up to P4000 grit size. The black spots on the BSE image represents surface contamination/oxidation and the visibly contrasted parallel lines are scratch marks from polishing (left panel). EDS mapping shows uniform distribution of Cu, Cr and Se across the sample (right panel).

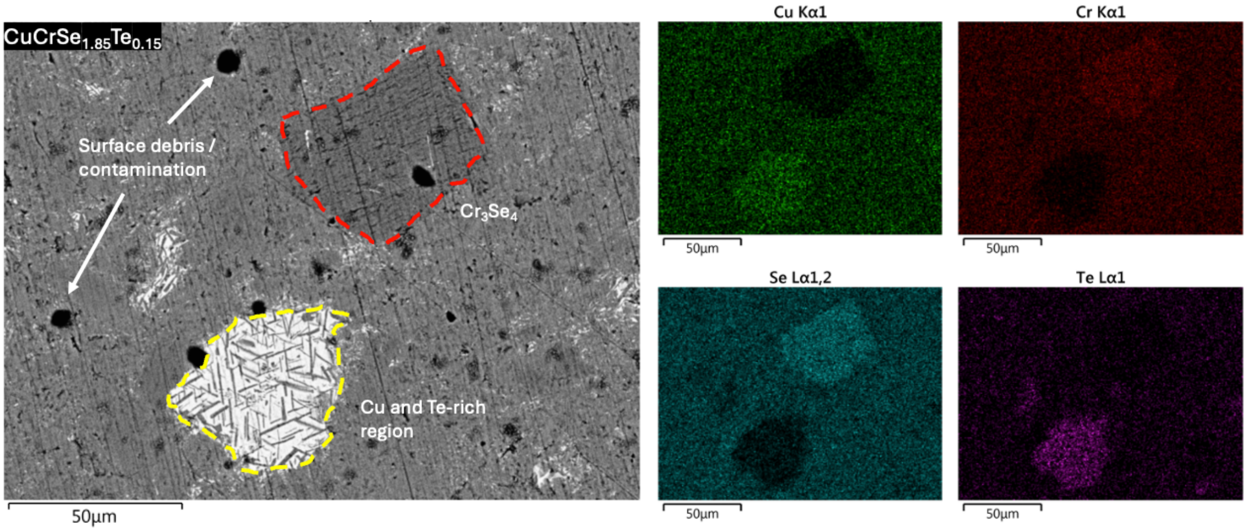

Figure S4: Backscattered electron (BSE) imaging of $CuCrSe_{1.85}Te_{0.15}$ sample, taken on flat parallel surface, polished up to P4000 grit size. The red marked region (dark grey) is secondary Cr_3Se_4 phase, and the yellow dashed line encloses Cu and Te-rich region. The black spots on the BSE image are surface debris (left panel). EDS mapping shows distribution of Cu, Cr and Se across the sample (right panel).

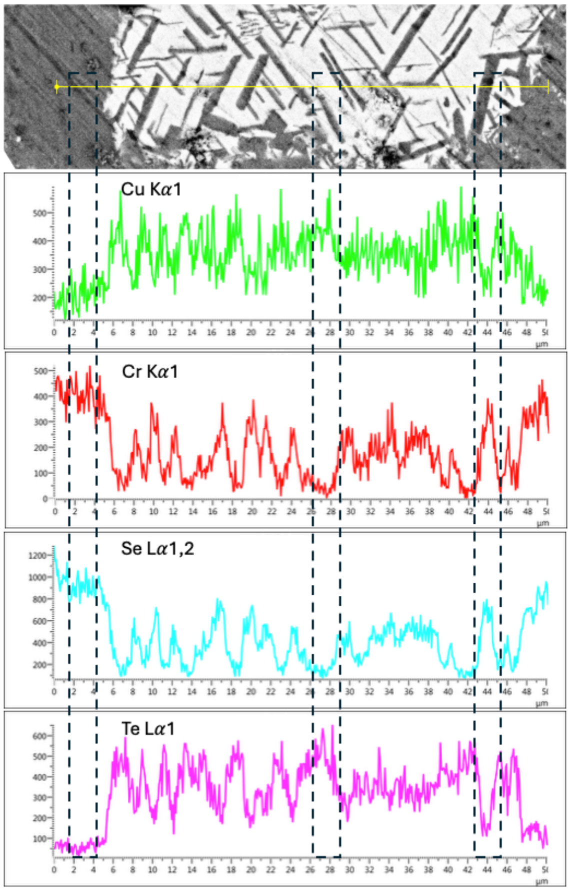

Figure S5: An EDS line scan across Cu and Te-rich area on the BSE image of $CuCrSe_{1.85}Te_{0.15}$ sample. Along the yellow line, intensity of Cu, Cr, Se and Te contents are plotted. The dashed rectangles are drawn to analyze 3 different zones: outside grey zone, white area and the inside grey area (from left to right). It appears that the white area is Cu and Te-rich with very low Cr and Se content, while the grey region is mostly $CuCrSe_{1.85}Te_{0.15}$ phase.

Variable temperature X-ray Diffraction

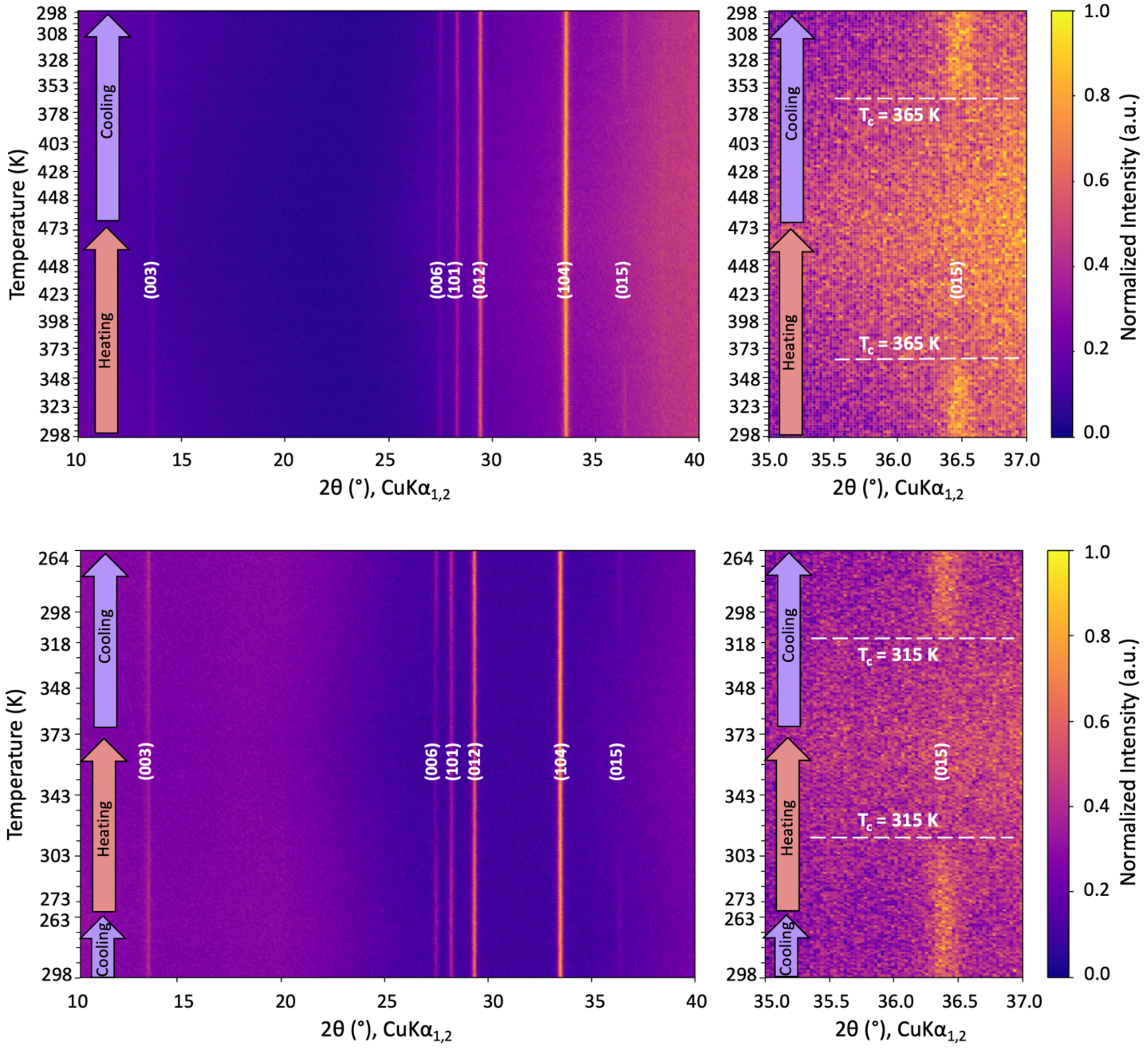

Figure S6: Contour plot of variable temperature X-ray diffraction for the $CuCrSe_2$ (top panel) and $CuCrSe_{1.9}Te_{0.1}$ (bottom panel) samples. In case of the $CuCrSe_2$ sample, the contrast in normalized intensity shows the appearance and disappearance of the (015) peak at 365 K, indicating a phase transition at that temperature. For *$CuCrSe_{1.9}Te_{0.1}$, the (015) peak disappears upon heating at 315 K and then reappears on cooling below the same temperature, indicating a phase transition temperature, T_c = 315 K.*

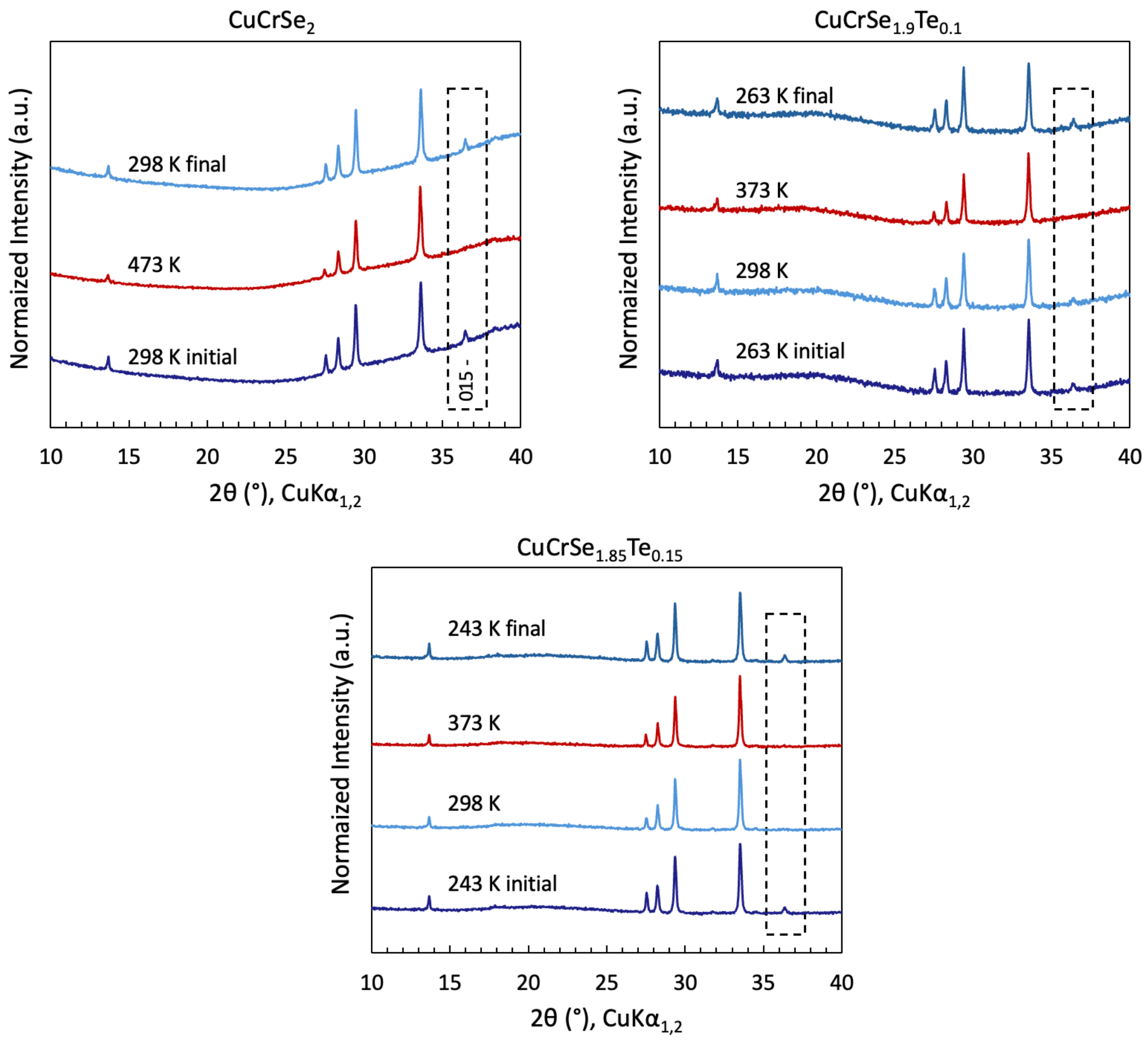

Figure S7: At predetermined temperature points, 15-minute X-ray diffraction patterns were collected for $CuCrSe_{2-x}Te_x$ samples at different temperatures. The (015) peak is observed to be present at some temperatures, which means the sample is in ordered phase at those temperatures. At temperatures, where the (015) peak is missing or heavily suppressed, the sample is in the superionic phase.

Thermal and Electronic Transport properties in $CuCrSe_{2-x}Te_x$

Figure S8(a) shows total thermal conductivity (κ_{total}) of $CuCrSe_{2-x}Te_x$ samples up to 500 K. The dashed lines are the experimental κ_{total} calculated using Duong-Petit approximation for C_p, while the markers are adjusted κ_{total} which subtracts the presumed latent heat absorbed during the phase transition [1]. The latter was used for calculations of zT, to ensure that the latent-heat dip does not cause an artificial peak in zT vs temperature. At room temperature, $CuCrSe_{1.9}Te_{0.1}$ and $CuCrSe_{1.85}Te_{0.15}$ exhibit significantly lower thermal conductivity (0.9 $Wm^{-1}K^{-1}$) than the $CuCrSe_2$ sample, while the differences in κ_{total} reduces after all the samples are in fully disordered phase.

Figure S8(b) and S8(c) show the electrical resistivity (ρ) and Seebeck coefficients (S) of the $CuCrSe_{2-x}Te_x$ compounds as a function of temperature. The phase transitions in $CuCrSe_2$ and $CuCrSe_{1.9}Te_{0.1}$ do not seem to cause any discontinuation in the plots. Electrical resistivity increases as Te is introduced in the system, reaching 3.09 mΩcm for $CuCrSe_{1.9}Te_{0.1}$ at room temperature. With more Te in the system the resistivity decreases to 2.04 mΩcm for $CuCrSe_{1.85}Te_{0.15}$ which is still higher than that of $CuCrSe_2$ (0.78 mΩcm). The positive Seebeck coefficients indicate hole-dominated transport agreeing with previous reports [2] on this material system. For all the samples, ρ and S increase with temperature, showing usual degenerate semiconductor behavior. Figure S8(d) shows that the thermoelectric figure of merit, $zT = S^2T/\rho\kappa$ [3,4], of the alloyed samples are lower than that of $CuCrSe_2$ at room temperature but eventually overlaps at above 400 K, with $CuCrSe_{1.85}Te_{0.15}$ reaching a maximum *zT* of 0.38 at 493 K.

The temperature-dependent Hall carrier concentration *(n_h),* shown in Figure S9(a), do not follow any particular trend with the amount of Te content in the samples, which can explain the similar random trend in electrical resistivity in the discussed compound series. With increasing temperature, n_h does not exhibit any significant change. The career concentration of $CuCrSe_{1.9}Te_{0.1}$ ($\approx 1.3\times 10^{20}$ cm^{-3}) is lower than that of $CuCrSe_2$ ($\approx 2\times 10^{20}$ cm^{-3}), but $CuCrSe_{1.9}Te_{0.1}$ was observed to have significantly higher n_h ($\approx 3\times 10^{20}$ cm^{-3}) than the rest of the samples. Unlike carrier concentration, the Hall mobility *(μ_h),* shown in Figure S9(b), decreases with increased amount of Te in the $CuCrSe_{2-x}Te_x$ series which can be attributed to reduced relaxation time as scattering increases.

Figure S10 shows the Seebeck coefficient, mobility, and *zT* as a function of carrier concentration (n_h). The single parabolic band model with the assumption of acoustic phonon scattering [5] was used for calculating the effective mass for $CuCrSe_2$ ($1.23m_0$) at 323 K and predicting the n_h dependence of the electronic transport properties. Fig S10(c) suggests that the best *zT* values for $CuCrSe_2$ can be achieved at n_h = 2.5×10^{19} cm^{-3}. Our experimental data on Seebeck and Hall mobility for $CuCrSe_{1.9}Te_{0.1}$ and $CuCrSe_{1.85}Te_{0.15}$ are also presented in Figure S10.

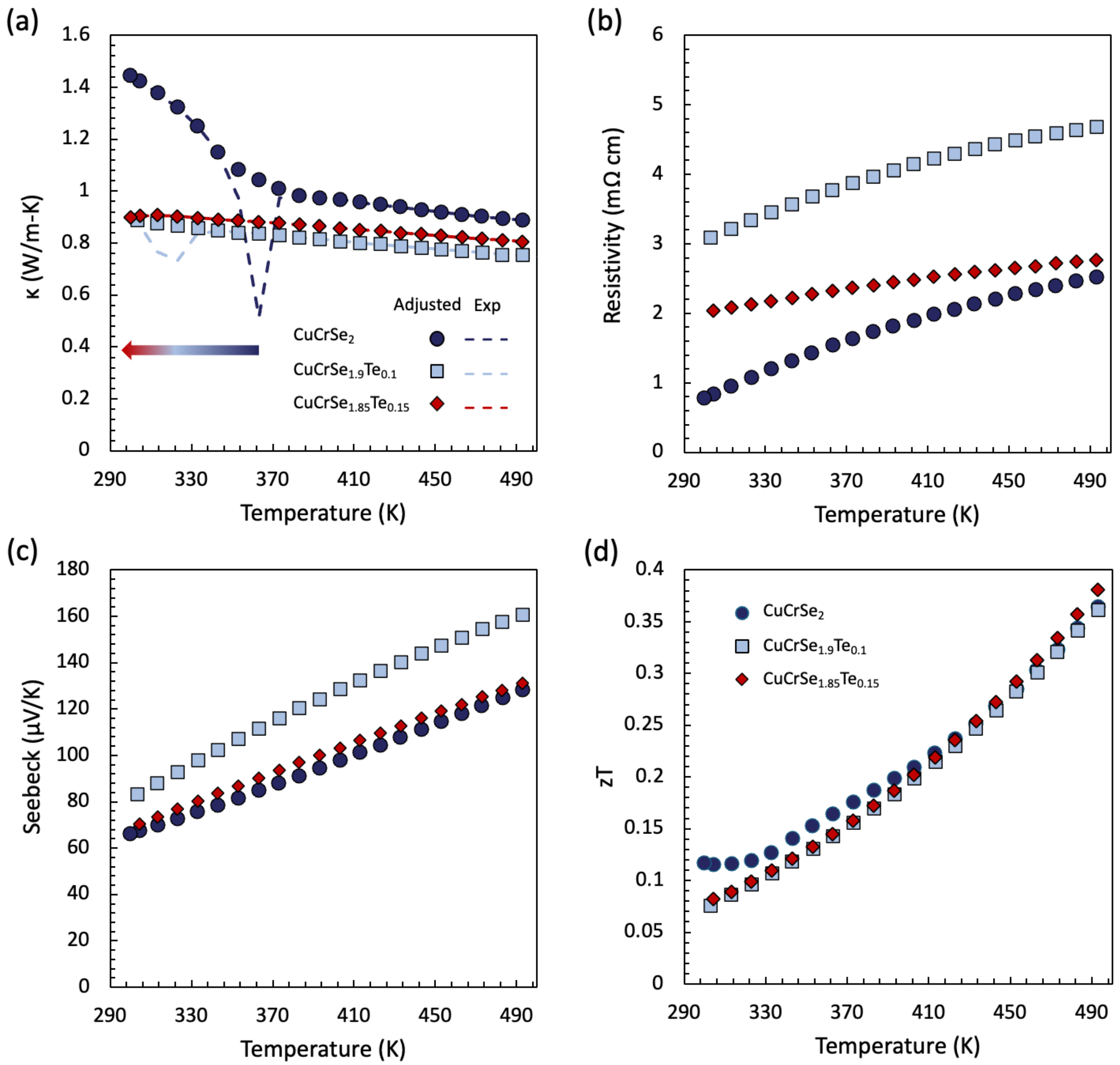

Figure S8: (a) Total thermal conductivity (κ_{total}) of $CuCrSe_{2-x}Te_x$ samples. The dashed lines are κ_{total} values obtained from thermal diffusivities using Dulong-Petit approximation and the symbol are adjusted κ_{total} values, in which we subtracted the presumed *latent heat. (b) Resistivity, (c) Seebeck coefficient and (d) thermoelectric figure of merit (zT) for the samples are shown here as a function of temperature. Data was collected on heating and cooling cycle, and the latter one is presented here.*

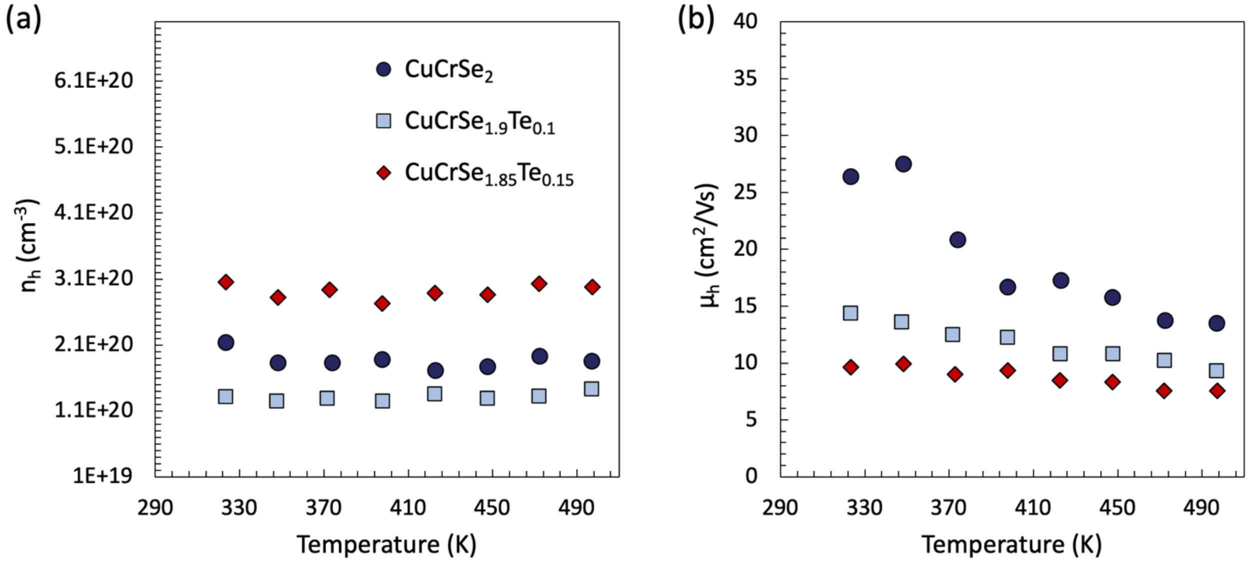

Figure S9: (a) Carrier concentration (n_h) and (b) Hall mobility (μ_h) of $CuCrSe_{2-x}Te_x$ samples. Mobility decreases as more Te is introduced at the anion sites, but no trend is observed in carrier concentration.

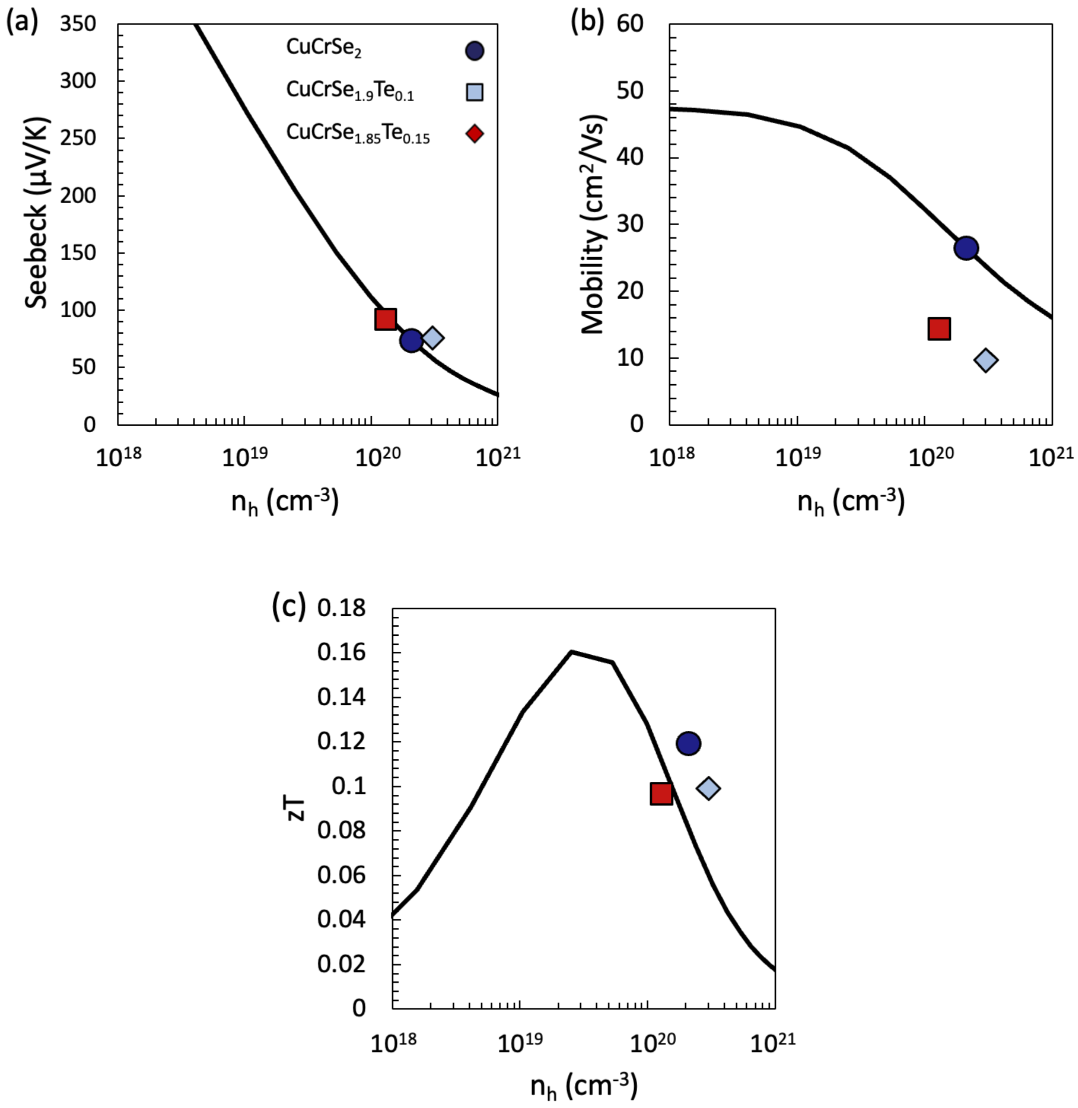

Figure S10: Pisarenko plot for (a) Seebeck coefficient, (b) Hall mobility, and (c) zT at 323 K for $CuCrSe_2$. The curves show the predictions within a single parabolic band model using effective mass of $1.23m_0$ and assuming that the dominant form of scattering is acoustic deformation potential scattering. This model suggests that a zT of 0.16 at 323 K can be achieved if the carrier concentration can be reduced to 2.5×10^{19} cm^{-3}.

Electronic and Lattice Thermal Conductivity in $CuCrSe_{2-x}Te_x$

Figure S11(a) shows electronic thermal conductivity, κ_e of the $CuCrSe_{2-x}Te_x$ compounds, which is calculated from the Wiedemann-Franz law, κ_e = LT/ρ, where L is the Lorenz number determined from Seebeck coefficient, L = 1.5 + exp (-|S| / 116) [6]. Figure S11(b) shows the temperature-dependent lattice thermal conductivity (κ_l) obtained from the difference between adjusted κ_{total} and κ_e data ($\kappa_l = \kappa_{total} - \kappa_e$). At room temperature, $CuCrSe_2$ has significantly larger electronic thermal conductivity ($\kappa_e \approx 0.8$ $Wm^{-1}K^{-1}$), compared to $CuCrSe_{1.9}Te_{0.1}$ (0.2 $Wm^{-1}K^{-1}$) and $CuCrSe_{1.85}Te_{0.15}$ (0.3 $Wm^{-1}K^{-1}$). At higher temperature, the difference among the electronic thermal conductivities is smaller. At room temperature, all of the samples show almost similar κ_l, ranging from 0.6 to 0.7 $Wm^{-1}K^{-1}$ and κ_l decreases slightly with increase in temperature. The unusual shape of κ_l in $CuCrSe_2$ is affected by the discontinuity in adjusted κ_{total} at the phase transition temperature.

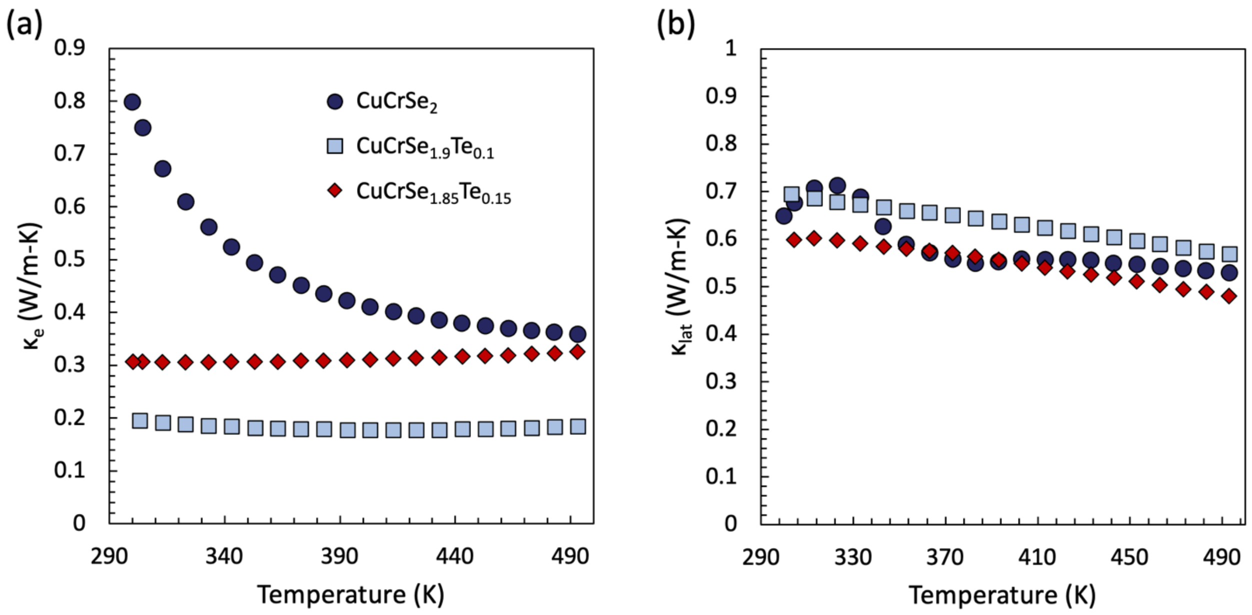

Figure 11: (a) Electronic thermal conductivity and (b) lattice thermal conductivity of $CuCrSe_{2-x}Te_x$ samples.

Transport Properties during Heating and Cooling Cycle in the $CuCrSe_{2-x}Te_x$ series

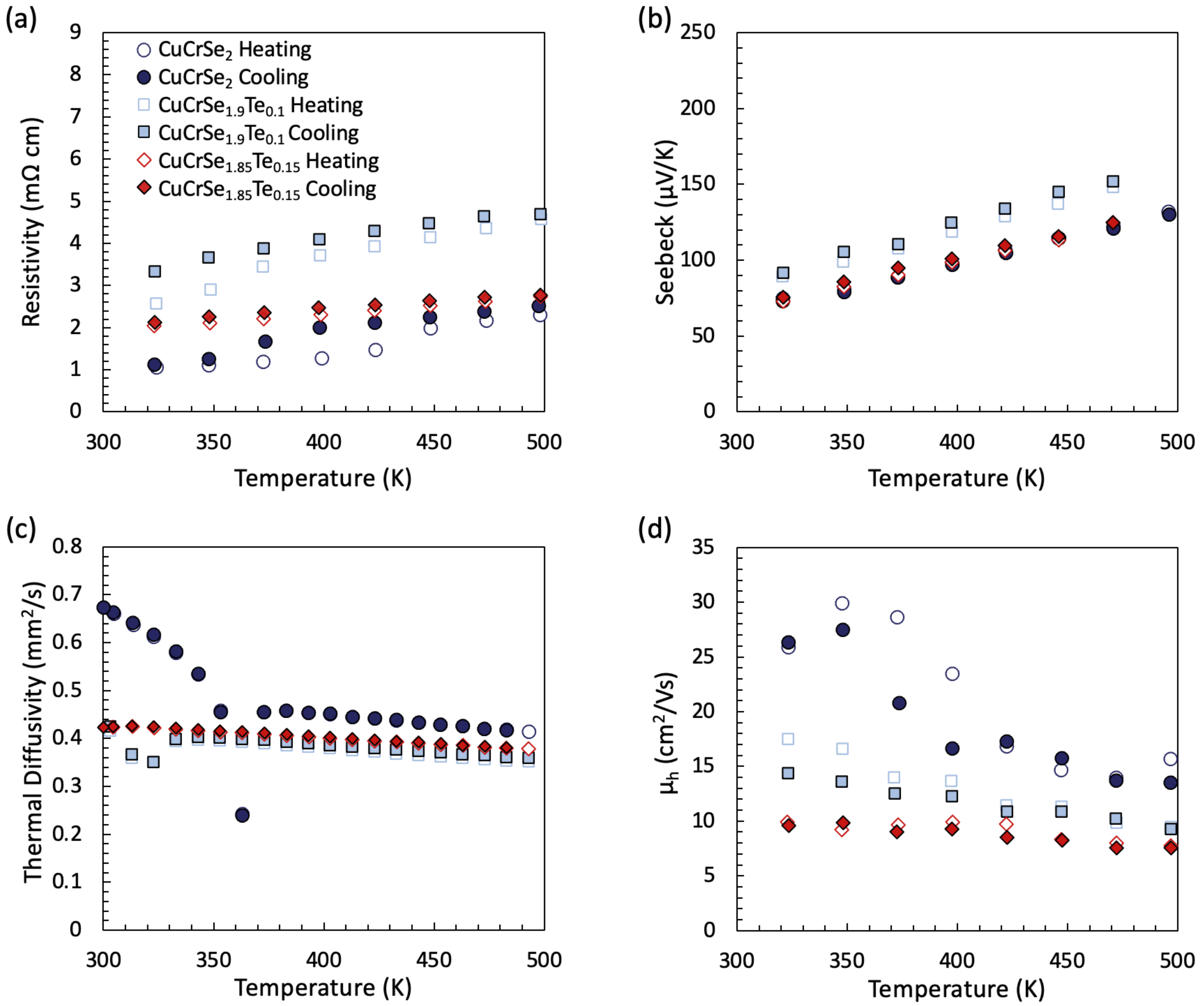

Figure S12: (a) Electrical resistivity, (b) Seebeck coefficient, (c) thermal diffusivity and (d) Hall mobility of $CuCrSe_{2-x}Te_x$ samples for heating and cooling cycles are shown here as a function of temperature.

Reproducibility of Electronic Transport Properties in the $CuCrSe_{2-x}Te_x$ series

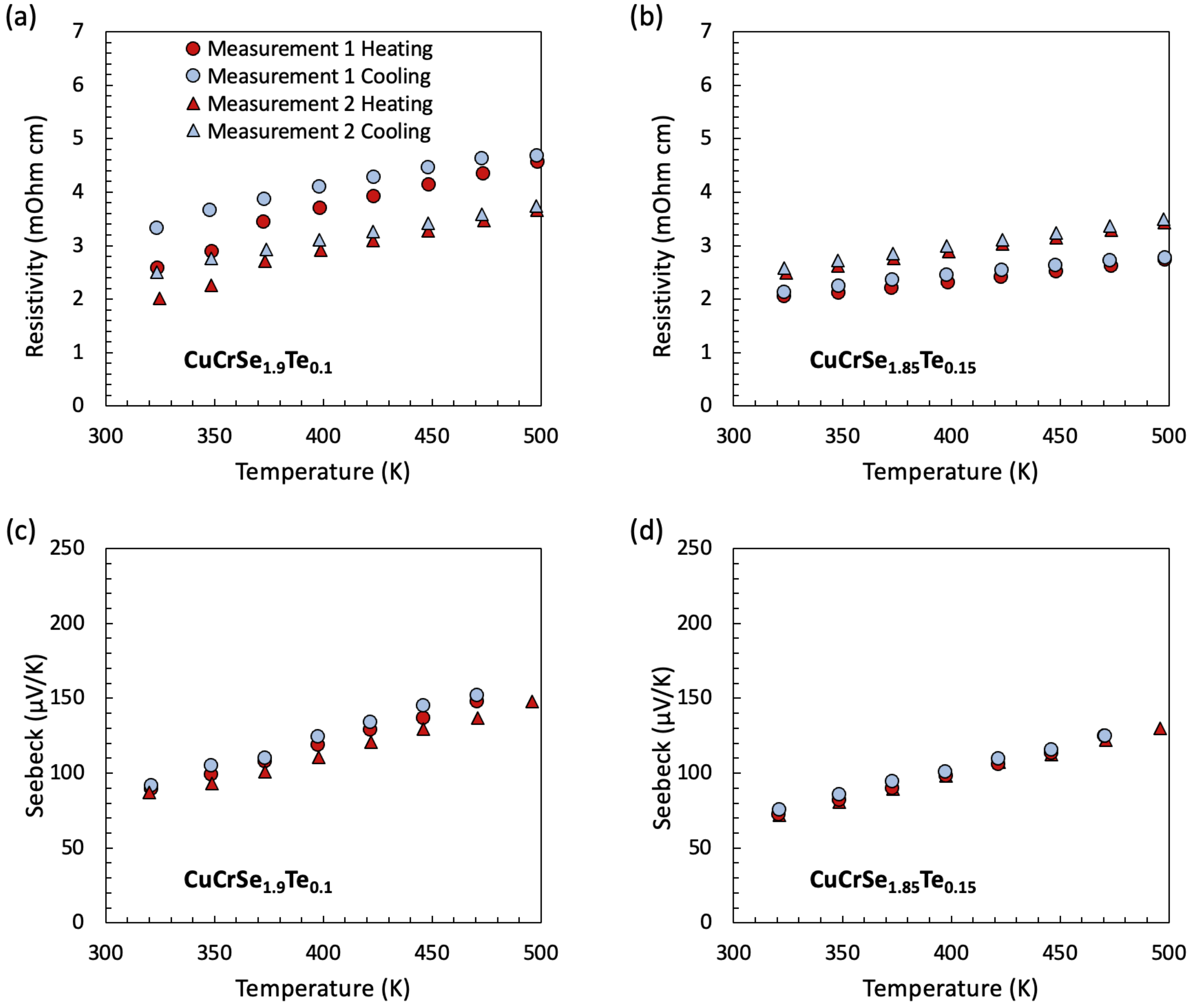

Figure S13: (a,b) Electrical resistivity and (c,d) Seebeck coefficient of $CuCrSe_{1.9}Te_{0.1}$ and $CuCrSe_{1.85}Te_{0.15}$ samples measured for multiple times.

Reproducibility and Stability Testing of Thermal Diffusivity in the $CuCrSe_{2-x}Te_x$ series

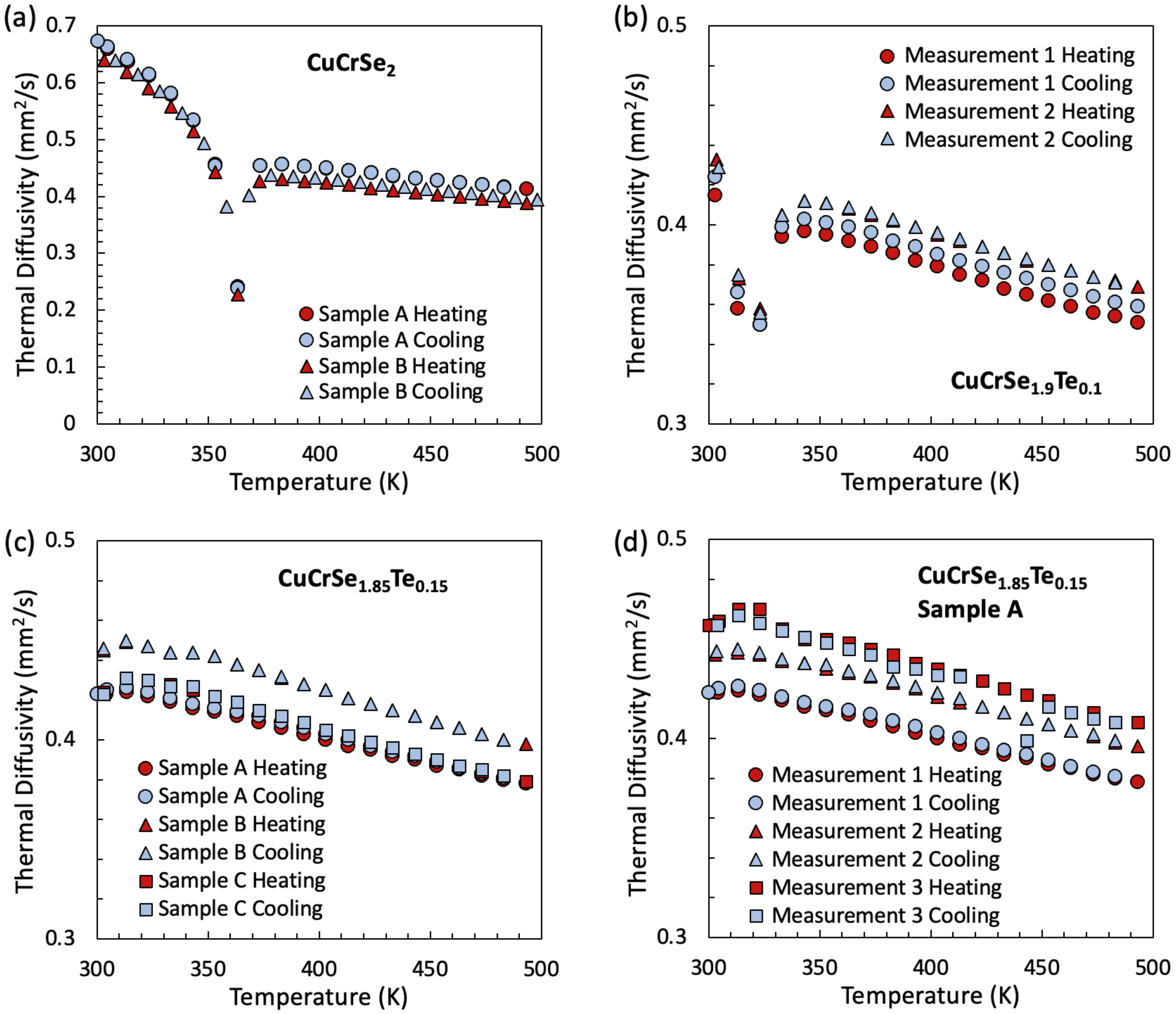

Figure S14: Thermal diffusivity measured on different samples of (a) $CuCrSe_2$, (b) $CuCrSe_{1.9}Te_{0.1}$ and (c,d) $CuCrSe_{1.85}Te_{0.15}$ compositions for multiple times.

Measurement of Elastic Properties during Heating Cycle in Multiple $CuCrSe_{2-x}Te_x$ Samples

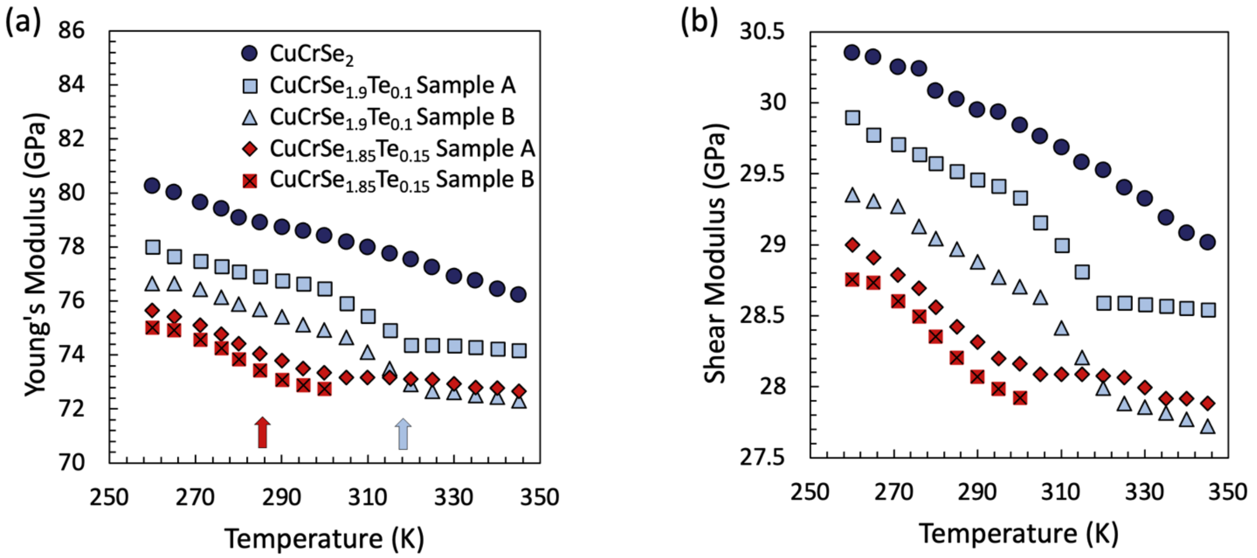

Figure S15: (a) Young's modulus and (b) shear modulus for $CuCrSe_{2-x}Te_x$ series measured during heating cycle on different samples for same composition.

Measurement of Elastic Properties during Heating and Cooling Cycles in the $CuCrSe_{2-x}Te_x$ series

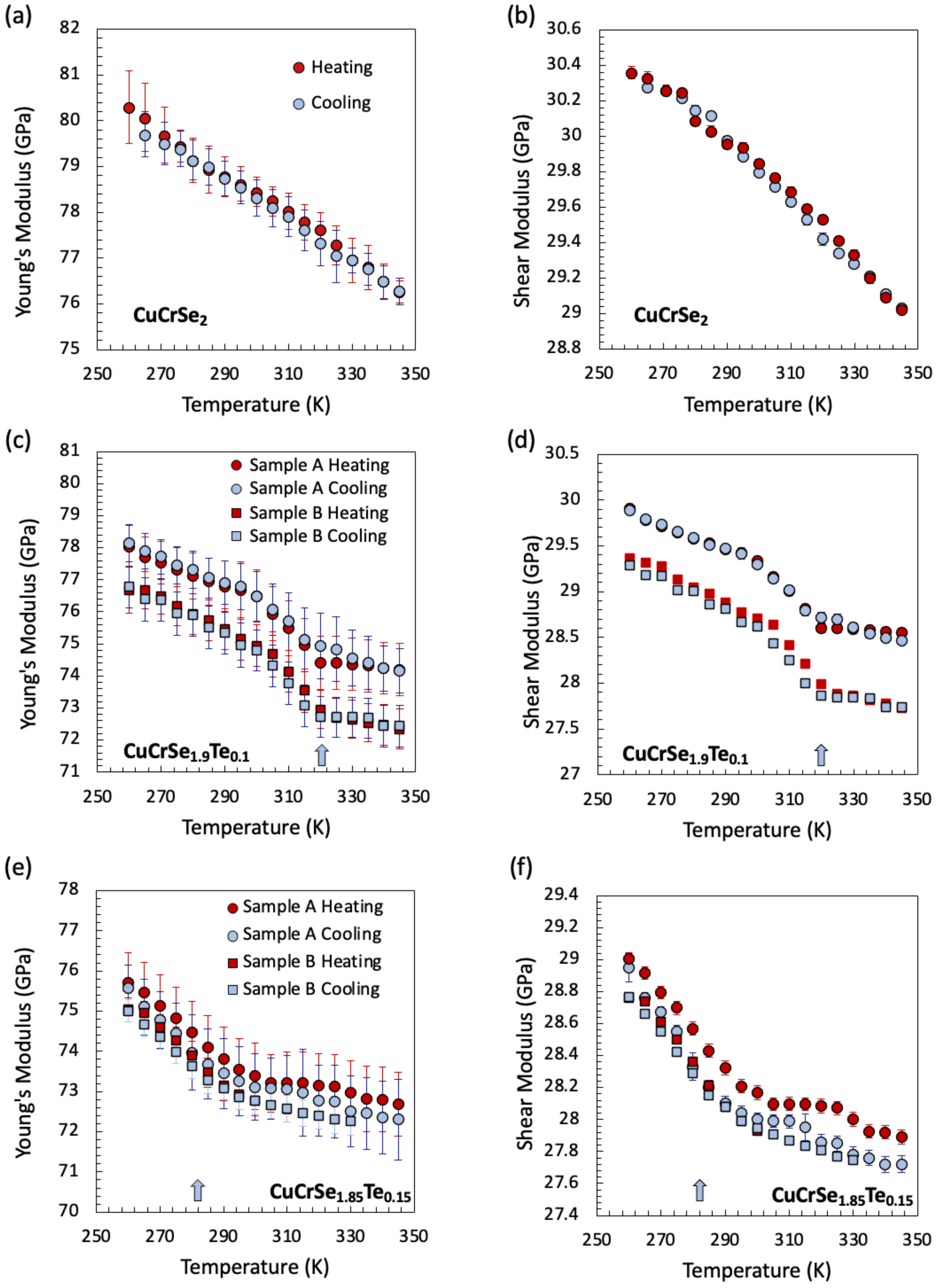

Figure S16: (a,c,e) Young's modulus and (b,d,f) shear modulus for $CuCrSe_2$ (one sample), $CuCrSe_{1.9}Te_{0.1}$ (multiple samples) and $CuCrSe_{1.85}Te_{0.15}$ (multiple samples) measured during heating and cooling cycles. Error bars are shown for individual data points, some of which are very small and hidden behind the data point symbols.

Equations for Elastic Properties

After the elastic tensor was solved, shear modulus (G) and bulk modulus (B) were obtained using equations discussed by Isotta *et al* [7]:

$$G = C_{44} \qquad B = C_{11} - \frac{4}{3}C_{44}$$

From the values of G and B, Young's modulus (Y), Poisson's ratio (μ), longitudinal velocity (v_L) and shear velocity (v_S) were calculated using the following equations [7], where ρ = geometric sample density,

$$Y = \frac{9BG}{3B+G} \qquad \mu = \frac{3B-2G}{2(3B+G)} \qquad v_S = \sqrt{\frac{G}{\rho}} \qquad v_L = \sqrt{\frac{B+\frac{4}{3}G}{\rho}}$$

Sample	C_{11}	C_{44}	B	Y	v_L (m/s)	v_S (m/s)	v_{avg} (m/s) (arithmetic)	v_{avg} (m/s) (cubic root)
$CuCrS_2$	133.08	37.05	83.68	96.86	5443	2872	3729	3211
$CuCrSe_{1.0}S_{1.0}$	116.77	32.80	73.16	85.60	4703	2492	3229	2785
$CuCrSe_{1.25}S_{0.75}$	113.91	32.14	71.06	83.79	4563	2424	3137	2709
$CuCrSe_{1.5}S_{0.5}$	110.88	30.90	69.68	80.76	4440	2344	3043	2620
$CuCrSe_{1.75}S_{0.25}$	113.38	30.94	72.12	81.21	4442	2310	3021	2585
$CuCrSe_{1.9}S_{0.1}$	105.47	29.91	65.59	77.89	4234	2255	2915	2519
$CuCrSe_2$	110.09	29.85	70.29	78.45	4336	2258	2951	2527
$CuCrSe_{1.9}Te_{0.1}$	104.01	29.34	64.89	76.49	4255	2260	2925	2525
$CuCrSe_{1.85}Te_{0.15}$	99.50	28.17	61.94	73.38	4111	2182	2825	2438

Table S1: Elastic properties and speed of sound for $CuCrSe_{2-y}S_y$ and $CuCrSe_{2-x}Te_x$ samples obtained by Resonance Ultrasound Spectroscopy (RUS) at 300 K.

References

[1] Rahman MT, Ciesielski K, Pelkey J, Shawon AKMA, Toberer E, Zevalkink A. Thermal and electronic transport properties of $ACrX_2$ superionic conductors (A=Cu, Ag and X=S, Se). Journal of Physics: Energy 2025;7:035016. https://doi.org/10.1088/2515-7655/addf7e.

[2] Bhattacharya S, Basu R, Bhatt R, Pitale S, Singh A, Aswal DK, Gupta SK, Navaneethan M, Hayakawa Y. $CuCrSe_2$: a high performance phonon glass and electron crystal thermoelectric material. J Mater Chem A Mater 2013;1:11289–94. https://doi.org/10.1039/C3TA11903C.

[3] Zevalkink A, Smiadak DM, Blackburn JL, Ferguson AJ, Chabinyc ML, Delaire O, Wang J, Kovnir K, Martin J, Schelhas LT, Sparks TD, Kang SD, Dylla MT, Snyder GJ, Ortiz BR, Toberer ES. A practical field guide to thermoelectrics: Fundamentals, synthesis, and characterization. Appl Phys Rev 2018;5. https://doi.org/10.1063/1.5021094.

[4] Shawon AKMA, Guetari W, Ciesielski K, Orenstein R, Qu J, Chanakian S, Rahman MT, Ertekin E, Toberer E, Zevalkink A. Alloying-Induced Structural Transition in the Promising Thermoelectric Compound CaAgSb. Chemistry of Materials 2024;36:1908–18. https://doi.org/10.1021/acs.chemmater.3c02621.

[5] Bux SK, Yeung MT, Toberer ES, Snyder GJ, Kaner RB, Fleurial J-P. Mechanochemical synthesis and thermoelectric properties of high quality magnesium silicide. J Mater Chem 2011;21:12259–66. https://doi.org/10.1039/C1JM10827A.

[6] Kim H-S, Gibbs ZM, Tang Y, Wang H, Snyder GJ. Characterization of Lorenz number with Seebeck coefficient measurement. APL Mater 2015;3:041506. https://doi.org/10.1063/1.4908244.

[7] Isotta E, Peng W, Balodhi A, Zevalkink A. Elastic Moduli: a Tool for Understanding Chemical Bonding and Thermal Transport in Thermoelectric Materials. Angewandte Chemie International Edition 2023;62:e202213649. https://doi.org/https://doi.org/10.1002/anie.202213649.
